# HECLIP: histology-enhanced contrastive learning for imputation of transcriptomics profiles

**DOI:** 10.1093/bioinformatics/btaf363

**Published:** 2025-06-26

**Authors:** Qing Wang, Wen-jie Chen, Jing Su, Guangyu Wang, Qianqian Song

**Affiliations:** Department of Health Outcomes and Biomedical Informatics, College of Medicine, University of Florida, Gainesville, FL 32611, United States; School of Biological and Behavioural Sciences, Queen Mary University of London, London, E1 4NS, United Kingdom; Department of Biostatistics and Health Data Science, Indiana University School of Medicine, Indianapolis, IN 46202, United States; Center for Bioinformatics and Computational Biology, Houston Methodist Research Institute, Houston, TX 77030, United States; Department of Cardiothoracic Surgery, Weill Cornell Medicine, Cornell University, Houston, NY, United States; Department of Health Outcomes and Biomedical Informatics, College of Medicine, University of Florida, Gainesville, FL 32611, United States

## Abstract

**Motivation:**

Histopathology, particularly hematoxylin and eosin (H&E) staining, is pivotal for diagnosing and characterizing pathological conditions by visualizing tissue morphology. However, H&E-stained images inherently lack molecular resolution, necessitating costly and labor-intensive technologies like spatial transcriptomics (ST) to uncover spatial gene expression patterns. There is a critical need for scalable computational methods that can bridge this imaging-transcriptomics gap.

**Results:**

We present histology-enhanced contrastive learning for imputation of profiles (HECLIP), an innovative deep learning framework designed to infer spatial gene expression profiles directly from H&E-stained histology images. HECLIP employs an image-centric contrastive learning strategy to capture morphological features relevant to molecular expression. By minimizing dependence on ST data, HECLIP enables accurate and biologically meaningful predictions of gene expression. Extensive benchmarking on publicly available datasets demonstrates that HECLIP outperforms existing methods. Ablation studies confirm the contribution of each model component to its overall performance.

**Availability and implementation:**

The source code for HECLIP is freely available at: https://github.com/QSong-github/HECLIP.

## 1 Introduction

Histopathology is widely recognized as a standard for identifying and characterizing diverse pathological conditions. Central to histopathological procedures is tissue staining, which differentiates intracellular components to facilitate visual interpretation. Among the staining techniques, hematoxylin and eosin (H&E) staining is the most widely used. It exploits the contrasting affinities of acidic eosin and basic hematoxylin dyes to highlight tissue morphology (Feldman and Wolfe 2014), providing pathologists with essential visual cues for diagnostic decision-making (Li *et al.* 2024). Despite its widespread use and diagnostic value, H&E-stained images inherently carry limited molecular information (Bonasia *et al.* 2015), necessitating the expertise of skilled pathologists to interpret nuanced features.

The spatial organization of gene expression within tissues plays a pivotal role in understanding complex biological processes and disease mechanisms. Spatial transcriptomics (ST) has emerged as a revolutionary technology that integrates spatial resolution with gene expression profiling, offering unparalleled insights into tissue heterogeneity and the microenvironment. However, the practical application of ST remains constrained by its high cost and specialized equipment requirements, which limit accessibility for routine clinical or research use (Wang *et al.* 2023b, Sharma *et al.* 2024). In contrast, histological imaging is an established and cost-effective technique capable of capturing tissue structure and morphology with high resolution. Computational approaches (Kolodziejczyk *et al.* 2015, Dorn *et al.* 2016) that infer spatial gene expression from histology images present a promising alternative to ST. These approaches bridge the gap between histology and transcriptomics by leveraging advanced deep learning models, providing a scalable and efficient solution that integrates molecular and morphological information, thereby advancing precision medicine (Rao *et al.* 2021, Zeng *et al.* 2022, Bai *et al.* 2024).

Recent studies have developed different methods to predict gene expression from histology images, demonstrating the potential of these computational approaches in reducing the reliance on expensive sequencing technologies for spatially resolved gene expression profiling. For instance, Contrastive Language-Image Pretraining (CLIP) (Radford *et al.* 2021) leverages contrastive learning to align image and text modalities, enabling applications such as cross-modal retrieval and classification. ST-Net (He *et al.* 2020) uses deep learning to predict local gene expression directly from H&E-stained images, while Bi-modaL Embedding for Expression Prediction (BLEEP) (Xie *et al.* 2024) employs a bi-modal embedding framework to map paired image and gene expression data into a unified embedding space. HisToGene (Pang *et al.* 2021), another advanced model, utilizes a Vision Transformer to capture spatial dependencies in histological data, improving gene expression predictions by integrating structural context.

Despite significant advancements, current methods still face limitations in accuracy and reliability. The accurate prediction of gene expression patterns from histological images is challenged by the biological complexity of tissues, where factors such as cell type and microenvironmental influences play critical roles. We summarize some classic methods in Table 17, available as supplementary data at *Bioinformatics* online.

In this paper, we present the HECLIP model, which leverages an innovative image-centric contrastive loss to optimize multimodal representation learning. By designing a tailored image-centric loss function, HECLIP enhances the representation capabilities of histological images, enabling more accurate predictions of transcriptomic data. This customized loss function is versatile and adaptable, making it applicable to a wide range of multimodal contrastive learning tasks. Extensive evaluations across multiple datasets demonstrate that HECLIP not only achieves robust performance but also consistently outperforms existing models in different datasets and scenarios, highlighting its effectiveness and broad applicability.

## 2 Materials and methods

**Figure 1.**
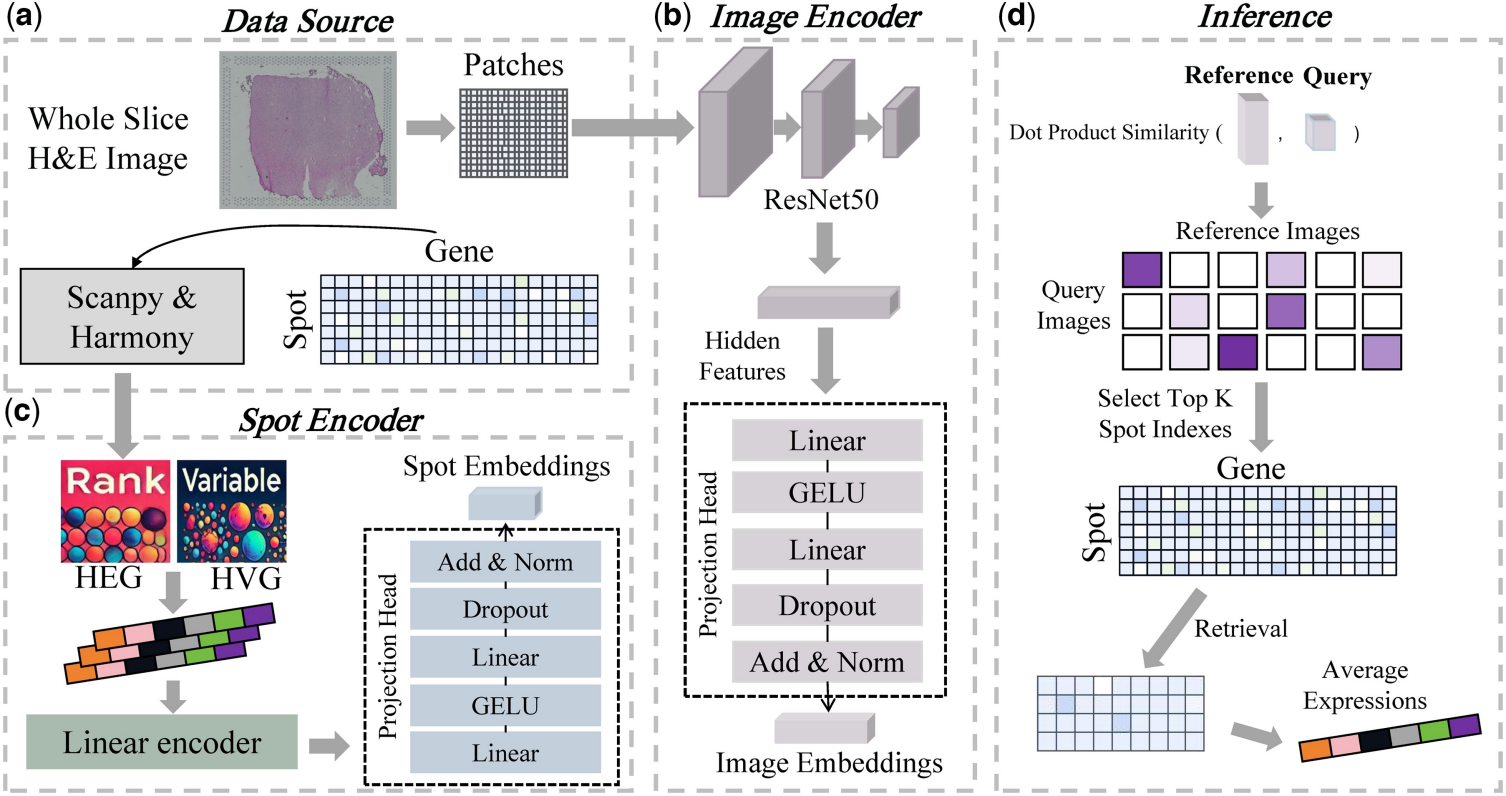
Overview of the HECLIP framework for transcriptomics imputation from histological images. (a) Data Source: Whole-slide H&E images are processed into patches, and gene expression data is obtained from spatial transcriptomics with Scanpy & Harmony. (b) Image Encoder: Patches are passed through a ResNet50-based encoder followed by a projection head to produce image embeddings. (c) Spot Encoder: Highly expressed genes (HEG) and highly variable genes (HVG) are selected and passed through a linear encoder and projection head to generate spot embeddings. (d) Inference: For a given query image, dot-product similarity is computed with reference images to select top-K most similar spots, and the corresponding gene expressions are averaged for imputation.

We present histology-enhanced contrastive learning for imputation of profiles (HECLIP), a deep learning framework (Fig. 1) designed to infer spatial gene expression profiles directly from hematoxylin and eosin (H&E)-stained histological images. Figure 1a illustrates the data preprocessing steps, where whole-slide histological images are divided into patches 256 × 256 pixels, and corresponding transcriptomic data are normalized and prepared using Scanpy. This preprocessing aligns histological image features with transcriptomic profiles (Gao *et al.* 2021, Yuan *et al.* 2023), enabling effective integration. For the image patches, the Image Encoder module (Fig. 1b) employs a ResNet-50 backbone combined with linear layers, GELU activations, normalization, and dropout layers to extract high-dimensional image embeddings. In parallel, transcriptomic data focusing on highly expressed genes (HEG) and highly variable genes (HVG) are processed by the Spot Encoder module (Fig. 1c) to generate transcriptomic embeddings at spatially resolved spots. These embeddings are aligned with the image features within a shared embedding space through contrastive learning, facilitating the seamless integration of molecular and morphological data. During the inference stage (Fig. 1d), embeddings from query image patches (e.g. new, unseen images) are compared to reference embeddings (e.g. training images) to identify the most relevant reference spots. The top-K similar spots are selected, and their gene expression profiles are retrieved and averaged to impute the spatial transcriptomic profiles for the query patches. In this way, HECLIP provides robust and biologically meaningful predictions, bridging the gap between histology and transcriptomics while offering a scalable and cost-effective alternative to traditional ST methods.

### 2.1 Datasets and preprocessing

To validate the effectiveness of HECLIP, we have evaluated its performance using the GSE240429 dataset (Andrews *et al.* 2024) (available at https://www.ncbi.nlm.nih.gov/geo/query/acc.cgi? acc=GSE240429), the GSE245620 dataset *(*Andrews *et al.* 2024) (available at https://www.ncbi.nlm.nih.gov/geo/query/acc.cgi? acc=GSE245620), and the spatialLIBD dataset (Maynard *et al.* 2021) (split into two datasets and available at https://research.libd.org/spatialLIBD/). These datasets, summarized in Table 1, are profiled using the 10× Genomics Visium platform.

**Table 1.** Details of datasets.

Datasets	Training size (reference)	Testing size (query)	Gene size
GSE240429_HVG	6992	2277	3467
GSE240429_HEG	6992	2277	3511
GSE245620_HVG	14 975	4992	3508
GSE245620_HEG	14 976	4992	3403
spatialLIBD_1_HVG	12 679	4789	3376
spatialLIBD_1_HEG	12 679	4789	3468
spatialLIBD_2_HVG	25 538	4110	3405
spatialLIBD_2_HEG	25 538	4110	3615

Gene size refers to the number of selected genes from their corresponding gene expression data.

The GSE240429 and GSE245620 datasets focus on the immunological status of healthy human livers and those affected by primary sclerosing cholangitis, a chronic liver disease characterized by bile duct inflammation and damage. Both datasets consist of four consecutive thick sections of human liver tissue from neurodegenerative donor livers deemed suitable for transplantation.

The spatialLIBD dataset is profiled with human dorsolateral prefrontal cortex slices, which includes regions spanning six neuronal layers plus white matter, covering three subjects with two pairs of spatially adjacent replicates per subject. This dataset comprises 12 slices in total, which were split into two subsets for our analysis: spatialLIBD 1 (4 slices) and spatialLIBD 2 (8 slices), based on sample continuity. Each slice covers all six cortical layers and white matter to ensure comprehensive spatial representation.

For each spot, we extract image patches from the whole-slide H&E stained histology images and paired them with corresponding gene expression profiles. Gene expression data are normalized and log-transformed using Scanpy (Wolf *et al.* 2018). Spot coordinates (x,y) are used to form patch–spot pairs, where the patch vertices are calculated as (x−128,y−128),(x−128,y+128),(x+128,y+128),(x+128,y−128), and (x+128,y−128), in pixels.

To evaluate HECLIP’s prediction capability across slices, we use one slice in every dataset as the test set (query), while the remaining slices are used for training (reference). For each spot, we test both HEG and HVG. For HEG, we rank genes in each spot based on expression levels, selecting the top 3500 genes across all spots. For HVG, we identify the most variable genes per slice, combine them across slices, and select 3500 genes for training and prediction. Batch effects between slices are corrected using the Harmonypy (Korsunsky *et al.* 2019) package to account for technical variability. This experimental setup enables a robust evaluation of HECLIP’s performance across diverse datasets.

### 2.2 Model input

Let $D=\{(p_{1},s_{1}),(p_{2},s_{2}),\ldots ,(p_{n},s_{n})\}$ represents the training dataset with *n* patch–spot pairs, where $p_{i}\in R^{256\times 256}$ is the input of image patch and $s_{i}\in R^{d}$ denotes the gene expression sequence. *d* refers to the number of HEG or HVG (gene size). We also use image data augmentation approach to improve data diversity. Specifically, we perform random flipping and rotations on each patch, which is commonly used in the visual field. However, we perform two random operations on a patch in one epoch, while the spot remains unchanged. Therefore, we get double the amount of data in each epoch. Ablation experiments are performed to verify the effectiveness of this strategy. We did not use paired data augmentation here because our experimental results showed that the effect of this augmentation was very poor under our loss setting (shown in Tables 9–11, available as supplementary data at *Bioinformatics* online).

### 2.3 Embedding modules

The image embedding module is the classic ResNet50 (He *et al.* 2016, Xie *et al.* 2017) model with a projection head. We use the pretrained ResNet50 weights from the “timm” package, while the projection head weights are randomly initialized. A batch of image patches is denoted as $P\in R^{|B|\times 256\times 256\times 3}$, where |B| represents the image batch size, 256 is the patch size, and 3 is the image channel. Specifically, ResNet50 consists of four residual block modules (Block1–Block4), each containing multiple convolution layers that further reduce the spatial size and increase the number of channels. Each residual block extracts more robust features through residual connections and convolution operations. First, through the processing of convolution layers and pooling layers, the dimensions (height and width) of images are gradually reduced, while the depth (number of channels) of image features are increased. Then the image features pass through the convolution blocks inside ResNet50. After the last convolution block, the feature map size obtained is (|B|,8,8,2048). The global average pooling layer performs average pooling on the 8×8 feature map on each channel and outputs an embedding of size (|B|). After the pooling operation, the final image representation $z_{p}\in R^{|B|\times 2048}$ (the shape is flattened) is obtained.

Projection Head consists of linear projection, nonlinear activation, layer normalization (LayerNorm), residual connection and dropout. First, there is a fully connected linear layer with the GELU activated function that maps the $h_{p}$ to embedding space. Next is a random dropout layer (dropout rate: 0.1) to relieve overfitting. Finally, there is a normalization layer to normalize the output and stabilize the training process. The formulas are as follows:


(1) $$h_{1}=\mathrm{GELU}(W_{1}z_{p}+b_{1})$$

(2) $$h_{2}=\mathrm{Dropout}(W_{2}h_{1}+b_{2})$$

(3) $$h_{p}=\mathrm{LayerNorm}(h_{1}+h_{2})$$

where $h_{p}\in R^{|B|\times d_{o}}$ is the image embedding and $d_{o}$ is the output dimension. For the embedding extraction module of spot data, considering its relatively low data complexity, we adopt a shallow linear network to embed the spots into the $d_{o}$ dimension, followed by a projection head similar to the image embedding module. Spot embedding can be expressed as $h_{s}\in R^{|B|\times d_{o}}$ with $d_{o}$ dimension of feature.

Algorithm 1 **Input:**

patch embedding ($h_{p}\in R^{|B|\times d_{o}}$)

spot embedding ($h_{s}\in R^{|B|\times d_{o}}$)

**Output:** loss value (l∈R*)*

1: **function**  cross_entropy(*logits*, *targets*)

2:   logits←LogSoftmax(logits, dim=-1)

3:   CE_loss←(−targets * logits).sum(1)

4:   **return** CE_loss

5: **end function**

6: **function**  image_centric_clip_loss($h_{p}$, $h_{s}$)

7:   $\text{logits}\leftarrow (h_{s}@h_{p}.T)/\text{temperature}$

8:   $\text{sim}\_\text{img}\leftarrow h_{p}@h_{p}.T$

9:   targets←sim_img/temperature

10:  targets←Softmax(targets)

11:  CE_loss←CROSS_ENTROPY(logits.T,targets.T)

12:  l←CE_loss.mean()

13:  **return** *l*

14: **end function**

### 2.4 Loss function

In this work, we have used an innovative contrastive learning loss in HECLIP, specifically tailored to optimize the model parameters for our unidirectional task. Traditional contrastive loss functions, as used in the CLIP model, balance the two modalities (e.g. images and text) equally during loss calculation, making them well suited for bidirectional tasks such as image-to-text or text-to-image mapping. However, our application focuses exclusively on predicting gene expression from histological images, a unidirectional objective. To address this, we prioritize the optimization of the image encoder in HECLIP, enhancing its ability to generate highly informative embeddings from histological images while aligning accurately with the corresponding spot profiles. We achieve this through a simple yet effective strategy: reducing or completely removing the impact of spot-based loss during training. Specifically, we implement an algorithm (detailed in Algorithm 1) that excludes spot-based loss from the training process. During training, 80% of the data are used for model training, while the remaining 20% are reserved for testing model performance. The model parameters associated with the lowest test loss are saved as the final configuration of the model. This approach allows the model to focus on refining the image encoder, ensuring accurate prediction of gene expression profiles solely from histological images.

### 2.5 Inference stage

During the inference phase, our approach diverges from the traditional CLIP (Radford *et al.* 2021) model, which retrieves similar samples from embeddings of opposite modalities (e.g. using image embeddings to match text embeddings). Instead, we focus on the optimized image encoder to exclusively utilize image modality embeddings for retrieval.

This process begins by extracting image embeddings from both the training and test data using the image encoder with fixed parameters, forming the reference set. Next, the test set images are input into the image encoder to generate the query set embeddings. For each query patch, we calculate the dot product similarity between its embedding with each embedding in the reference set, ranking the results by similarity scores.

A predefined value of *K* is then used to select the *K* most similar reference patches for each query patch. The corresponding labels of the selected reference patches are retrieved, and the predicted gene expressions for the query patches are determined by averaging the gene expression profiles of these *K* reference patches. This approach leverages the optimized image encoder to ensure accurate and robust predictions based on the similarity of image embeddings.

### 2.6 Evaluation metrics

We have used several popular evaluation metrics in the experiments, including root mean square error (RMSE) and structural similarity index (SSIM) (Li *et al.* 2022).

RMSE measures the deviation between the predicted gene expressions and the actual gene expressions within each spot. The smaller the RMSE, the better the prediction performance of the model.


(4) $$\mathrm{RMSE}=\sqrt{\frac{1}{M}\sum_{j=1}^{M}{\left({\tilde{e}}_{ij}-e_{ij}\right)}^{2}}$$

where $e_{ij}$ and ${\tilde{e}}_{ij}$ are the normalized spatial expression of gene *i* in spot *j* in the ground truth and the predicted result, respectively. SSIM measures the similarity between predicted and true gene expressions across spots. The value of SSIM ranges between −1 and 1. SSIM values closer to 1 indicates more accurate predictions. Following the procedures of Li *et al.* (2022), we scaled the expression matrix as follows:


(5) $$e_{ij}^{\prime }=\frac{e_{ij}}{\max\left(\left\{e_{i1},\ldots ,e_{iM}\right\}\right)}$$

where $e_{ij}$ denotes the expression of gene *i* in spot *j*, and *M* is the total number of spots. Then, we calculate the SSIM value as follows:


(6) $$\mathrm{SSIM}=\frac{\left(2{\tilde{u}}_{i}u_{i}+C_{1}^{2}\right)\left(2\text{cov}\left(e_{i}^{\prime },{\tilde{e}}_{i}^{\prime }\right)+C_{2}^{2}\right)}{\left({\tilde{u}}_{i}^{2}+u_{i}^{2}+C_{1}^{2}\right)\left({\tilde{\sigma }}_{i}^{2}+\sigma_{i}^{2}+C_{2}^{2}\right)}$$

where $\mu_{i}$ and ${\tilde{u}}_{i}$ are the average expression value of gene *i* in the ground truth and the predicted result, respectively; and $\sigma_{i}$ and ${\tilde{\sigma }}_{i}$ are the s.d. of the ground truth and the predicted result, respectively. cov(·) is the covariance. The $C_{1}$ and $C_{2}$ are small constants to stabilize the calculation. We also used the top gene hit rate Hit@T  *(*inspired by extreme multilabel classification; Wang *et al.* 2023a)). Hit@*T* is defined as follows: (i) the gene with the highest predicted expression is selected; (ii) the first *K* genes from the ground truth (ranked from highest to lowest expression) are considered; (iii) if the predicted gene appears within these *K* ground truth genes, it is classified as correct; otherwise, it is classified as incorrect.


$$\mathrm{Hit}@T=\frac{1}{N}\sum_{i=1}^{N}I\left({\;\text{pred}\;}_{i}\cap {\;\text{true}\;}_{i}\neq \emptyset \right),$$

(7*)*where *I* is the indicator function, which equals 1 if the intersection of the two sets is nonempty (i.e. if there is at least one common index) and 0 otherwise. *T* specifies the selection range, for example, if *T*** **=** **3, it means selecting the top 3 highest-expressed genes and checking the overlap between the predicted top-expressed genes and the actual top-expressed genes.

## 3 Results

### 3.1 Benchmarking experiments demonstrate superior performance of HECLIP

**Figure 2.**
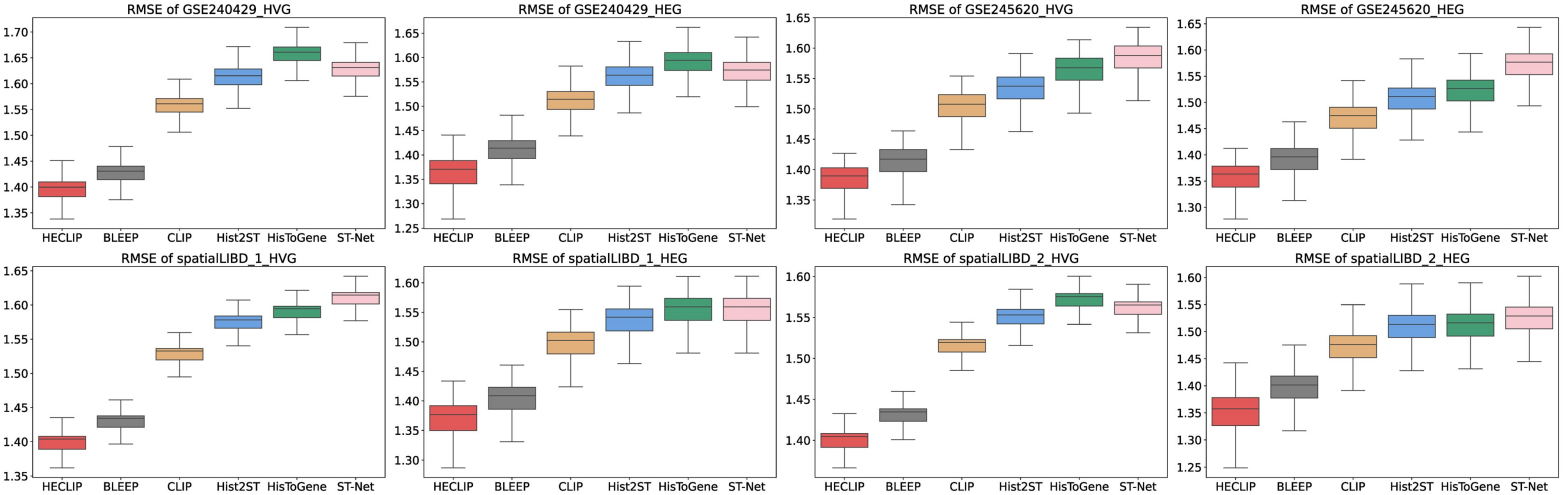
Comparison of methods for predicting transcriptomics from histology images based on RMSE metrics.

To evaluate the performance of HECLIP compared to existing methods, we conduct benchmarking experiments on multiple publicly available datasets (see Section 2.1). The benchmarking results demonstrate that HECLIP consistently outperforms other methods across all datasets in both HVG zand HEG scenarios, as evidenced by the SSIM (Fig. 1, Table 4, available as supplementary data at *Bioinformatics* online), RMSE (Fig. 2, Table 3, available as supplementary data at *Bioinformatics* online), Pearson correlation coefficient (PCC) (Table 2, available as supplementary data at *Bioinformatics* online) metrics. Specifically, in the GSE240429_HVG and GSE245620_HVG datasets, the median RMSE values for HECLIP were 1.40 and 1.39, respectively, with corresponding mean RMSE of 1.39 and 1.37, outperforming other models. For the SSIM metric, HECLIP also excels, achieving a median SSIM of 0.007 and of 0.011 in the GSE240429_HVG and GSE245620_HVG dataset, higher than BLEEP and CLIP. This trend is also observed in the spatialLIBD datasets. For example, in spatialLIBD_2_HEG, HECLIP achieved a median SSIM of 0.0285 and a mean SSIM of 0.048. Moreover, HECLIP exhibited lower variability in performance, particularly in datasets such as GSE240429_HEG and spatialLIBD_2_HEG, as shown in the box plots for RMSE and SSIM. This indicates that HECLIP is not only more accurate but also more stable and reliable. In contrast, other models like BLEEP and CLIP demonstrated lower SSIM values, particularly in datasets such as spatialLIBD_2, while HisToGene and ST-Net showed consistently poor predictive performance overall.

The results for Hit@*T* (*T* = 1, 2, 3) are presented in Table 2 (Hit@*T*, *T* = 1, 2, 3, 4, 5, are shown in Tables 1 and 5, available as supplementary data at *Bioinformatics* online). HECLIP consistently achieved the highest accuracy across all Hit@*T* values, clearly demonstrating its superior predictive capability. For instance, on the spatialLIBD_1_HVG dataset, HECLIP’s Hit@1 reached 0.37, significantly outperforming BLEEP (0.26), CLIP (0.21), HisToGene (0.17), and ST-Net (0.16). Similarly, on spatialLIBD_2_HVG, HECLIP achieved a remarkable Hit@1 of 0.60, surpassing BLEEP (0.38), CLIP (0.37), HisToGene (0.33), and ST-Net (0.36). Moreover, as the *T* value increased, the Hit@*T* values improve for HECLIP. Taken together, these benchmarking results demonstrate the robustness and superior predictive capability of HECLIP compared to alternative approaches. In addition, the PCCs are shown in Tables 2 and 6, available as supplementary data at *Bioinformatics* online.

**Table 2.** Comparative analysis of experimental results for Hit@T metrics.

	GSE240429_HVG			GSE240429_HEG			GSE245620_HVG			GSE245620_HEG		
	Hit@3	Hit@2	Hit@1	Hit@3	Hit@2	Hit@1	Hit@3	Hit@2	Hit@1	Hit@3	Hit@2	Hit@1
HECLIP	**0.9873**	**0.9627**	**0.8208**	**1**	**1**	**0.9978**	**0.9942**	**0.9772**	**0.8634**	**0.9998**	**0.999**	**0.9926**
BLEEP	0.9838	0.957	0.796	**1**	**1**	**0.9978**	0.9914	0.9637	0.8253	**0.9998**	**0.999**	**0.9926**
CLIP	0.9527	0.9113	0.7667	**1**	0.9923	0.9516	0.917	0.9003	0.7551	0.9536	0.9433	0.9177
Hist2ST	0.9423	0.9075	0.7362	0.9673	0.9331	0.9008	0.8763	0.8431	0.4602	0.8812	0.8397	0.5556
HisToGene	0.9399	0.8669	0.6723	0.9637	0.9191	0.8367	0.8679	0.8458	0.4135	0.8777	0.859	0.5327
ST-Net	0.9188	0.8657	0.6568	0.9618	0.9208	0.8468	0.8703	0.8431	0.4469	0.8712	0.8466	0.5612

	spatialLIBD_1_HVG			spatialLIBD_1_HEG			spatialLIBD_2_HVG			spatialLIBD_2_HEG		
	Hit@3	Hit@2	Hit@1	Hit@3	Hit@2	Hit@1	Hit@3	Hit@2	Hit@1	Hit@3	Hit@2	Hit@1
HECLIP	**0.8839**	**0.688**	**0.3688**	**0.9994**	**0.9793**	**0.5999**	**0.9987**	**0.976**	**0.5968**	**0.9993**	**0.9917**	**0.6214**
BLEEP	0.8321	0.6636	0.2585	0.9987	0.976	0.5368	0.9039	0.7088	0.3787	0.9987	0.976	0.5968
CLIP	0.8689	0.8593	0.2102	0.9796	0.964	0.496	0.8991	0.6987	0.3655	0.9789	0.9692	0.5551
Hist2ST	0.8118	0.7779	0.1799	0.883	0.8627	0.4411	0.8996	0.6561	0.3527	0.8863	0.8311	0.5271
HisToGene	0.8019	0.7758	0.1713	0.8816	0.8615	0.4398	0.873	0.6456	0.3333	0.8439	0.8009	0.5178
ST-Net	0.8113	0.7963	0.1628	0.9013	0.8688	0.4513	0.8695	0.6428	0.3618	0.8415	0.7998	0.5231

Bold font indicates the best value.

### 3.2 Loss convergence and embedding visualization

**Figure 3.**
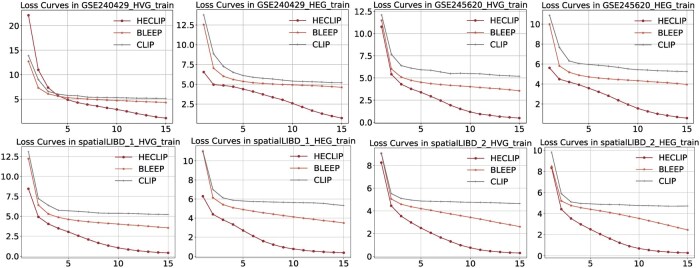
Comparison of loss convergence across different methods in training stage.

Figure 3 shows the reduction of loss over 15 epochs for different methods in training stage (testing stage is shown in Fig. 2, available as supplementary data at *Bioinformatics* online). HECLIP, optimized with an image-centric contrastive loss function, consistently outperforms both BLEEP and CLIP that rely on conventional loss functions. This advantage is evident in both training and test loss, highlighting the effectiveness of our tailored contrastive loss in enhancing model optimization and predictive capabilities.

**Figure 4.**
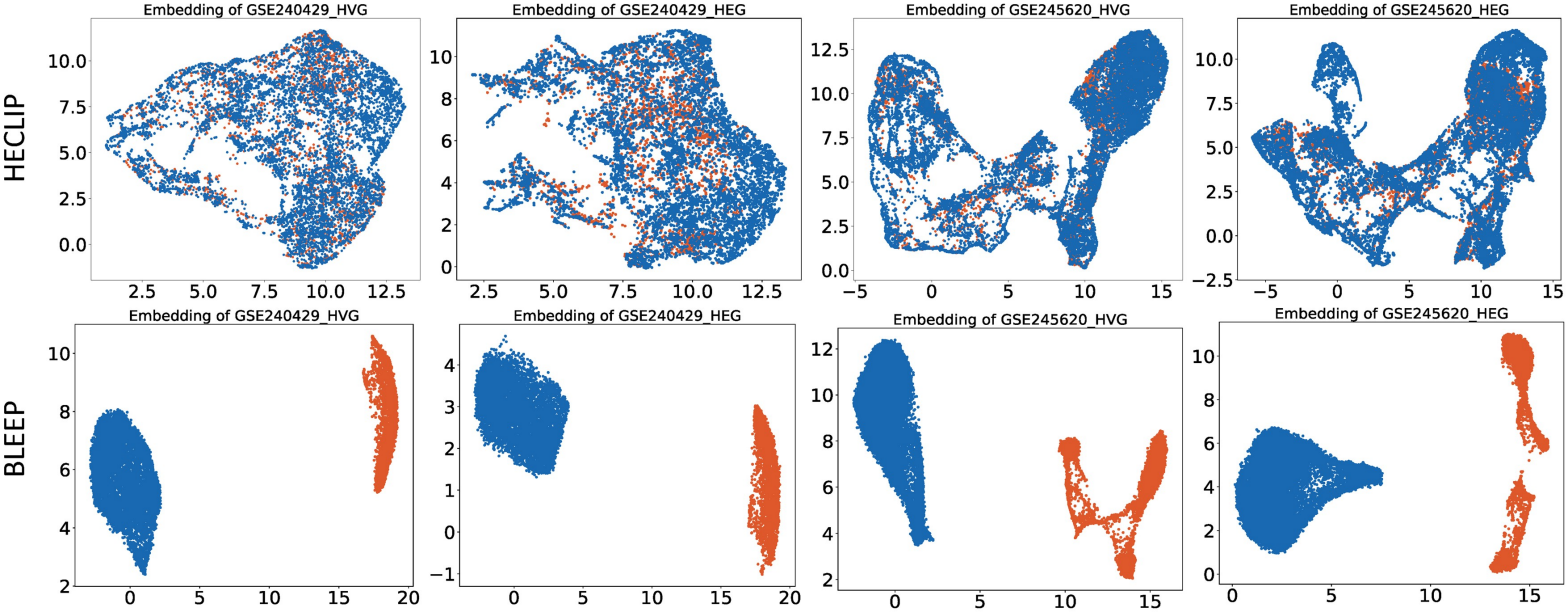
UMAP of the bi-modality embeddings from HECLIP and BLEEP. The blue dots are the Reference set and the orange dots are the Query set.

Additionally, UMAP visualizations of the embeddings in GSE240429 and GSE245620 generated by HECLIP and BLEEP are presented in Fig. 4 (spatialLIBD is shown in Fig. 3, available as supplementary data at *Bioinformatics* online), revealing significant differences in clustering patterns among the methods. The primary goal of these embeddings is to retrieve patches from the reference set that closely match those in the query set, which requires well-mixed and coherent representations. For BLEEP, the embeddings of the query and reference sets appear scattered, with limited integration between the two, indicating suboptimal alignment. In contrast, HECLIP’s embeddings exhibit a more cohesive and compact clustering, effectively mixing the reference and query sets. This demonstrates HECLIP’s ability to accurately capture similar patches. This cohesive embedding pattern is consistently observed across different datasets, underscoring the model’s robustness and reliability. These findings highlight the effectiveness of HECLIP’s unimodal contrastive loss in generating well-mixed, biologically meaningful embeddings, which significantly contribute to its superior overall performance.

### 3.3 HECLIP accurately predicts biologically important genes

To evaluate whether HECLIP uniquely identifies biologically meaningful genes, we conducted a comparative analysis of the top 200 genes ranked by PCC across all models. Notably, HECLIP uniquely prioritized several functionally important genes, including EIPR1 and PPIAL4A, which were not well predicted by any of the other methods (BLEEP, CLIP, Hist2ST, HisToGene, or ST-Net). For instance, HECLIP achieved PCC scores of 0.54 for EIPR1 and 0.44 for PPIAL4A, whereas all other models yielded PCC values below 0.1 for these genes, indicating little to no predictive capability. PPIAL4A, a member of the cyclophilin family, is involved in protein folding, isomerization, and immune modulation. Cyclophilins are associated with liver inflammation and fibrosis, hallmark features of chronic liver diseases like cirrhosis and Hepatocellular carcinoma (HCC). Cyclophilin inhibitors have shown promise in mitigating liver fibrosis and inflammation, underscoring their therapeutic potential (Naoumov 2014). Additionally, EIPR1 regulates endoplasmic reticulum (ER) stress, a process triggered by misfolded or unfolded protein accumulation. ER stress is a major contributor to liver diseases such as nonalcoholic fatty liver disease and HCC (Chen *et al.* 2023). Moreover, HECLIP consistently outperformed competing models on well-established liver markers such as CYP3A4 and GLUL. For CYP3A4, HECLIP achieved a PCC of 0.76, outperforming BLEEP (0.74), CLIP (0.68), Hist2ST (0.54), HisToGene (0.54), and ST-Net (0.55). For GLUL, HECLIP scored 0.66, again exceeding the performance of BLEEP (0.64), CLIP (0.58), Hist2ST (0.41), HisToGene (0.46), and ST-Net (0.46). These results demonstrate that HECLIP not only improves overall predictive accuracy but also enhances biomarker discovery.

### 3.4 Ablation experiments of the HECLIP model

In the ablation experiment, the effectiveness of HECLIP is thoroughly validated, as evidenced by the RMSE (Tables 7 and 11, available as supplementary data at *Bioinformatics* online), SSIM (Table 3, Table 11, available as supplementary data at *Bioinformatics* online), PCC (Tables 8 and 9, available as supplementary data at *Bioinformatics* online), and Hit@T (Table 10, available as supplementary data at *Bioinformatics* online) results presented. As shown in the tables, the HECLIP model consistently achieves relatively stable experimental outcomes in both w/o loss and w/o data settings.

**Table 3.** Ablation experiment results of SSIM on all datasets.

SSIM ↑	Median	Mean	Median	Mean
	GSE240429_HVG		GSE240429_HEG	
HECLIP	**0.007049**	**0.01889**	**0.02382**	**0.04594**
w/o loss	0.005791	0.01753	0.02192	0.04419
w/o data	0.005993	0.01681	0.02241	0.04444
	GSE245620_HVG		GSE245620_HEG	
HECLIP	**0.01105**	**0.02737**	**0.02537**	**0.04421**
w/o loss	0.00996	0.02641	0.02287	0.04322
w/o data	0.01012	0.02322	0.02391	0.04326
	spatialLIBD_1_HVG		spatialLIBD_1_HEG	
HECLIP	**0.008819**	**0.03224**	**0.01877**	**0.03452**
w/o loss	0.007891	0.03178	0.01819	0.03318
w/o data	0.007911	0.03	0.01796	0.03268
	spatialLIBD_2_HVG		spatialLIBD_2_HEG	
HECLIP	**0.008607**	**0.0309**	**0.02854**	**0.04782**
w/o loss	0.007928	0.02858	0.02815	0.04631
w/o data	0.007872	0.02511	0.02801	0.04474

Bold font indicates the best value.

w/o loss refers to the use of the original CLIP loss function. When using the original CLIP loss function, the model’s performance on SSIM and RMSE is slightly worse compared to the HECLIP’s image centric loss function. Specifically, for the GSE240429_HVG dataset, the RMSE median and mean values are 1.41 and 1.40, respectively, while the SSIM median and mean values are 0.006 and 0.018. Similarly, in the spatialLIBD_2_HEG dataset, the RMSE median and mean values are 1.36 and 1.35, and the SSIM median and mean values are 0.028 and 0.046, respectively. While these metrics are lower than those achieved using the improved loss function, they remain superior to other methods such as ST-Net, highlighting the reliability of the improved loss function. This conclusion is further supported by the loss convergence in Fig. 3.

w/o data indicates that no data augmentation strategy is employed. When data augmentation is not utilized, the model’s performance on RMSE and SSIM is also worse. For instance, in the GSE240429_HEG dataset, the RMSE median and mean values are 1.3882 and 1.3656, and the SSIM median and mean values are 0.02241 and 0.04444. Similarly, in the spatialLIBD_2_HVG dataset, the RMSE median and mean values are 1.4201 and 1.4047, and the SSIM median and mean values are 0.007872 and 0.02511.

Notably, employing both data augmentation techniques and the image centric loss function simultaneously yields the best performance. For example, in the GSE245620_HVG dataset, the RMSE median and mean values achieved by HECLIP are 1.3898 and 1.3743, while the SSIM median and mean values are 0.01105 and 0.02737, respectively. These results underscore the synergistic benefits of integrating the image centric loss function with data augmentation, achieving superior predictive performance and stability across various datasets. We have also performed experiments of hyperparameter tuning, please refer to Tables 12, 13, and 14, available as supplementary data at *Bioinformatics* online, for the results.

## 4 Discussion

This paper introduces HECLIP, an innovative CLIP-based model equipped with a specially designed unimodal contrastive loss function to enhance the representation capability of histological images. HECLIP exhibits strong scalability and adaptability, making it highly effective for contrastive learning tasks that align images with gene expression profiles. HECLIP performs especially well on datasets with well-defined tissue architecture and complex spatial expression gradients. Comprehensive experiments across diverse datasets demonstrate that HECLIP consistently outperforms state-of-the-art models, delivering superior predictions with exceptional robustness and reliability.

## Supplementary Material

btaf363_Supplementary_Data

## Data Availability

The data underlying this article are available in the article and in its online supplementary material.
