# Supplementary material for: HECLIP: histology-enhanced contrastive learning for imputation of transcriptomics profiles
Source: Bioinformatics. 2025 Jun 26;41(7):btaf363. doi: 10.1093/bioinformatics/btaf363 (PMC12362354; doi:10.1093/bioinformatics/btaf363)
Supplement: btaf363_Supplementary_Data [file btaf363_supplementary_data.zip › Clean Supplementary Material.pdf]

Supplementary Material for HECLIP: Histology-Enhanced Contrastive Learning for Imputation of Transcriptomics Profiles

Contents

A Experimental results **1**

B Ablation study **8**

C Hyperparameter study **12**

D Cross dataset analysis **14**

E Summary of conventional methods **14**

F Division of the training and testing datasets **15**

G Experimental design for CLIP **16**

A Experimental results

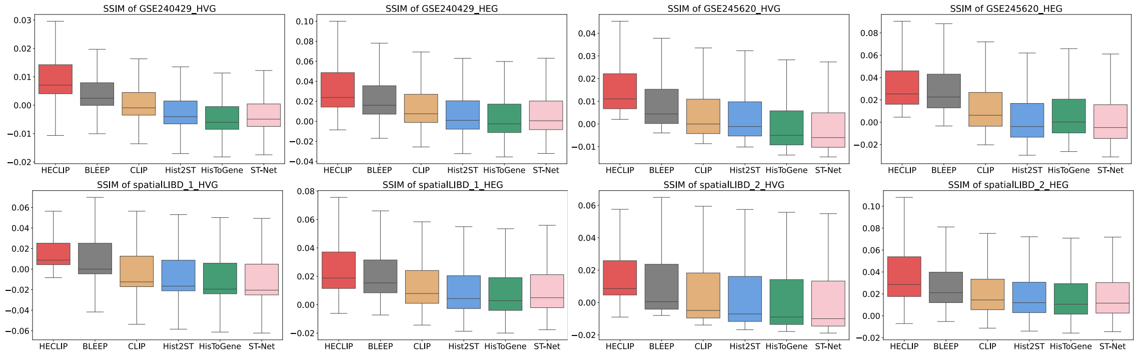

Figure S1: Comparison of methods for predicting transcriptomics from histology images based on SSIM metrics.

For the SSIM metric, HECLIP achieved a median SSIM of 0.007 and of 0.011 in the GSE240429_HVG and GSE245620_HVG dataset, higher than BLEEP and CLIP. This trend is also observed in the spatialLIBD datasets. For example, in spatialLIBD_2_HEG, HECLIP achieved a median SSIM of 0.0285 and a mean SSIM of 0.048. Moreover, HECLIP exhibited lower variability in performance, particularly in datasets such as GSE240429_HEG and spatialLIBD_2_HEG, as shown in the boxplots for RMSE and SSIM. This indicates that HECLIP is not only more accurate but also more stable and reliable. In contrast, other models like BLEEP and CLIP demonstrated lower SSIM values, particularly in datasets such as spatialLIBD_2, while HisToGene and ST-Net showed consistently poor predictive performance overall.

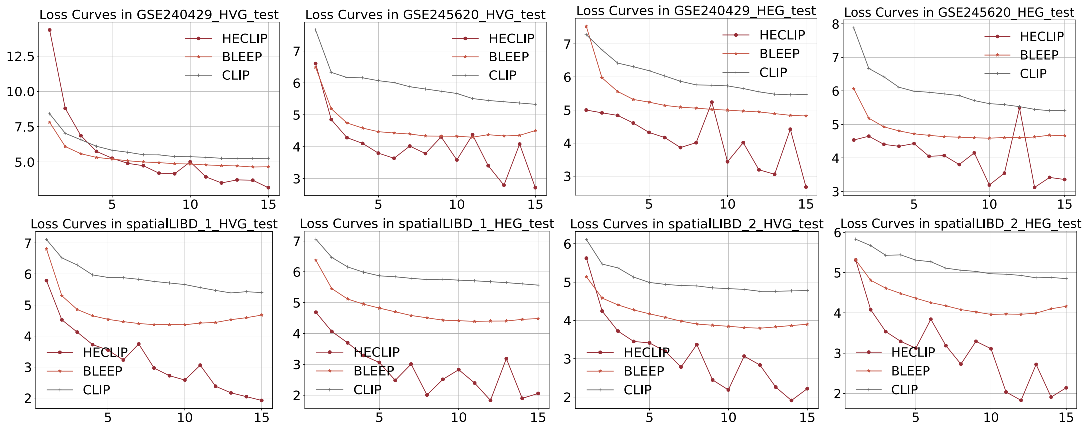

Figure S2: Comparison of loss convergence across different methods in testing stage.

Figure S2 shows the reduction of loss over 15 epochs for different methods in testing stage. HECLIP, optimized with an image-centric contrastive loss function, consistently outperforms both BLEEP and CLIP that rely on conventional loss functions. This advantage is evident in both training and test loss, highlighting the effectiveness of our tailored contrastive loss in enhancing model optimization and predictive capabilities.

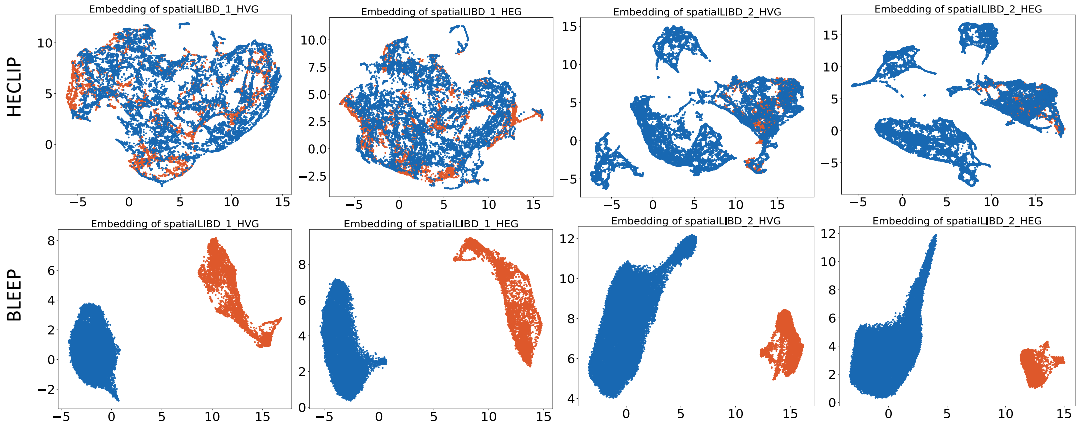

Figure S3: UMAP of the bi-modality embeddings from HECLIP and BLEEP. The blue dots are the Reference set and the orange dots are the Query set.

UMAP visualizations of the embeddings in spatialLIBD_1 and spatialLIBD_2 generated by HECLIP and BLEEP are presented in Figure S3, revealing significant differences in clustering patterns among the methods. The primary goal of these embeddings is to retrieve patches from the reference set that closely match those in the query set, which requires well-mixed and coherent representations. For BLEEP, the embeddings of the query and reference sets appear scattered, with limited integration between the two, indicating suboptimal alignment. In contrast, HECLIP's embeddings exhibit a more cohesive and compact clustering, effectively mixing the reference and query sets. This demonstrates HECLIP's ability to accurately capture similar patches. This cohesive embedding pattern is consistently observed across different datasets, underscoring the model's robustness and reliability. These findings highlight the effectiveness of HECLIP's unimodal contrastive loss in generating well-mixed, biologically meaningful embeddings, which significantly contribute to its superior overall performance.

We performed a Wilcoxon test on the Hit@K index of all comparison methods and ablation models on all datasets, as shown in Figure S4. In general, the Wilcoxon test results show the difference in performance between HECLIP and other methods. In each subplot, the dashed lower triangle is the Wilcoxon statistic and the dashed upper triangle is the corresponding p-value. For example, from the results of GSE240429_HVG, it can be seen that HECLIP has higher Wilcoxon statistic values (such as 5.50, 3.00, 5.00) in comparison with various comparison methods (such as w/o loss, w/o data, BLEEP, etc.), and the corresponding p-values are all 0.062, which is close to the commonly used significance threshold of

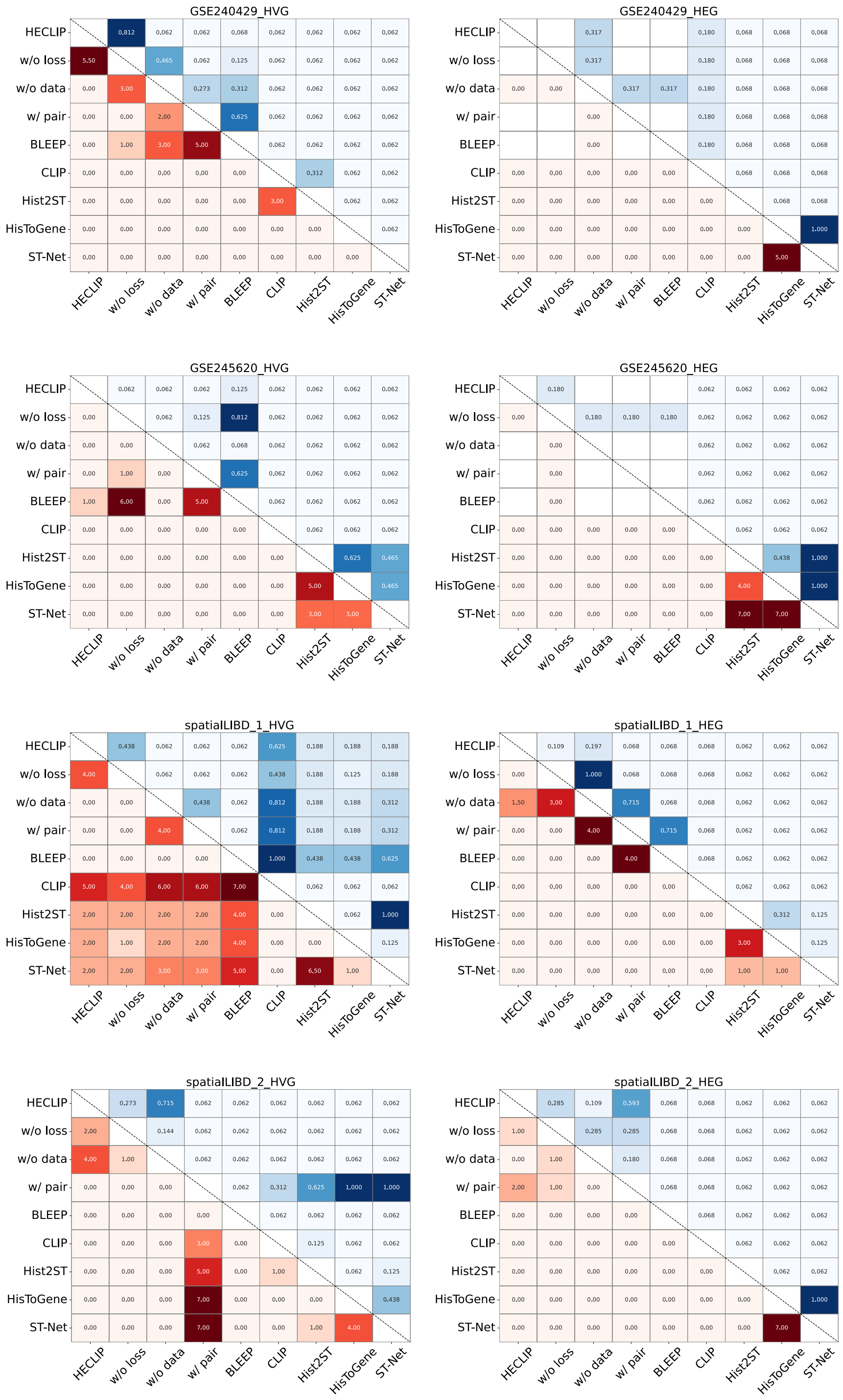

Figure S4: Wilcoxon test results, where the blank part indicates that the comparison data are consistent. In each subplot, the dashed lower triangle is the Wilcoxon statistic and the dashed upper triangle is the corresponding p-value.

0.05. This shows that HECLIP shows strong discrimination ability in multiple comparisons, and the differences are potentially statistically significant, indicating that this method has better performance than other models.

	**GSE240429_HVG**					**GSE240429_HEG**				
	**Hit@5**	**Hit@4**	**Hit@3**	**Hit@2**	**Hit@1**	**Hit@5**	**Hit@4**	**Hit@3**	**Hit@2**	**Hit@1**
**HECLIP**	**0.9982**	**0.9952**	**0.9873**	**0.9627**	**0.8208**	**1**	**1**	**1**	**1**	**0.9978**
**BLEEP**	**0.9982**	0.9947	0.9838	0.957	0.796	**1**	**1**	**1**	**1**	**0.9978**
**CLIP**	0.9786	0.9713	0.9527	0.9113	0.7667	**1**	**1**	**1**	0.9923	0.9516
**Hist2ST**	0.9865	0.9678	0.9423	0.9075	0.7362	**1**	0.9899	0.9673	0.9331	0.9008
**HisToGene**	0.9531	0.9466	0.9399	0.8669	0.6723	**1**	0.9737	0.9637	0.9191	0.8367
**ST-Net**	0.9469	0.9416	0.9188	0.8657	0.6568	**1**	0.9718	0.9618	0.9208	0.8468
	**GSE245620_HVG**					**GSE245620_HEG**				
	**Hit@5**	**Hit@4**	**Hit@3**	**Hit@2**	**Hit@1**	**Hit@5**	**Hit@4**	**Hit@3**	**Hit@2**	**Hit@1**
**HECLIP**	0.9978	**0.9968**	**0.9942**	**0.9772**	**0.8634**	**0.9998**	**0.9998**	**0.9998**	**0.999**	**0.9926**
**BLEEP**	**0.9984**	0.9962	0.9914	0.9637	0.8253	**0.9998**	**0.9998**	**0.9998**	**0.999**	**0.9926**
**CLIP**	0.9677	0.9301	0.917	0.9003	0.7551	0.9873	0.9873	0.9536	0.9433	0.9177
**Hist2ST**	0.9107	0.8997	0.8763	0.8431	0.4602	0.9315	0.9088	0.8812	0.8397	0.5556
**HisToGene**	0.9122	0.8993	0.8679	0.8458	0.4135	0.9311	0.9011	0.8777	0.859	0.5327
**ST-Net**	0.9122	0.9018	0.8703	0.8431	0.4469	0.9298	0.9076	0.8712	0.8466	0.5612
	**spatialLIBD_1_HVG**					**spatialLIBD_1_HEG**				
	**Hit@5**	**Hit@4**	**Hit@3**	**Hit@2**	**Hit@1**	**Hit@5**	**Hit@4**	**Hit@3**	**Hit@2**	**Hit@1**
**HECLIP**	**0.9712**	**0.9463**	**0.8839**	**0.688**	**0.3688**	**1**	**0.9998**	**0.9994**	**0.9793**	**0.5999**
**BLEEP**	0.9584	0.9181	0.8321	0.6636	0.2585	**1**	0.9996	0.9987	0.976	0.5368
**CLIP**	0.9112	0.8847	0.8689	0.8593	0.2102	**1**	0.9877	0.9796	0.964	0.496
**Hist2ST**	0.8407	0.8256	0.8118	0.7779	0.1799	**0.9518**	0.9303	0.883	0.8627	0.4411
**HisToGene**	0.8315	0.8119	0.8019	0.7758	0.1713	0.9528	0.9311	0.8816	0.8615	0.4398
**ST-Net**	0.8412	0.8239	0.8113	0.7963	0.1628	0.9499	0.9377	0.9013	0.8688	0.4513
	**spatialLIBD_2_HVG**					**spatialLIBD_2_HEG**				
	**Hit@5**	**Hit@4**	**Hit@3**	**Hit@2**	**Hit@1**	**Hit@5**	**Hit@4**	**Hit@3**	**Hit@2**	**Hit@1**
**HECLIP**	**1**	**0.9996**	**0.9987**	**0.976**	**0.5968**	**1**	**1**	**0.9993**	**0.9917**	**0.6214**
**BLEEP**	0.9859	0.9669	0.9039	0.7088	0.3787	**1**	0.9996	0.9987	0.976	0.5968
**CLIP**	0.9671	0.9409	0.8991	0.6987	0.3655	**1**	0.9981	0.9789	0.9692	0.5551
**Hist2ST**	0.9339	0.9218	0.8996	0.6561	0.3527	0.9527	0.9014	0.8863	0.8311	0.5271
**HisToGene**	0.9002	0.8819	0.873	0.6456	0.3333	0.9332	0.8867	0.8439	0.8009	0.5178
**ST-Net**	0.9112	0.8852	0.8695	0.6428	0.3618	0.9405	0.8753	0.8415	0.7998	0.5231

Table S1: Experiment results of Hit@T on all datasets.

The results for Hit@T (T=1,2,3,4,5) are presented in Table S1. HECLIP consistently achieved the highest accuracy across all Hit@T values, clearly demonstrating its superior predictive capability. For instance, on the spatialLIBD_1_HVG dataset, HECLIP's Hit@1 reached 0.37, significantly outperforming BLEEP (0.26), CLIP (0.21), HisToGene (0.17), and ST-Net (0.16). Similarly, on spatialLIBD_2_HVG, HECLIP achieved a remarkable Hit@1 of 0.60, surpassing BLEEP (0.38), CLIP (0.37), HisToGene (0.33), and ST-Net (0.36). In spatialLIBD_2_HVG, HECLIP's Hit@5 and Hit@4 reached 1 and 0.9996 respectively, which are higher than BLEEP's 0.9859 and 0.9669 and the performance of other models. In general, Hit@4 and Hit@5 have not increased much compared to Hit@3. From the perspective of model comparison, HECLIP is the best.

	GSE240429_HVG		GSE240429_HEG		GSE245620_HVG		GSE245620_HEG	
	**HEG**	**HVG**	**HEG**	**HVG**	**HEG**	**HVG**	**HEG**	**HVG**
**HECLIP**	**0.2766**	**0.2124**	**0.255**	**0.1933**	**0.2753**	**0.263**	**0.4297**	**0.2289**
**BLEEP**	0.1751	0.1712	0.084	0.1653	0.2535	0.2429	0.4016	0.2155
**CLIP**	0.1235	0.1217	0.0562	0.1235	0.1512	0.1977	0.3134	0.1682
**HIist2ST**	0.0973	0.1005	0.0416	0.1195	0.1107	0.1556	0.2681	0.1231
**HisToGene**	0.0527	0.0766	0.0321	0.0885	0.0725	0.1142	0.2082	0.0795
**ST-Net**	0.0983	0.0896	0.031	0.0916	0.1004	0.1203	0.1763	0.0977
	**spatialLIBD_1_HVG**		**spatialLIBD_1_HEG**		**spatialLIBD_2_HVG**		**spatialLIBD_2_HEG**	
	**HEG**	**HVG**	**HEG**	**HVG**	**HEG**	**HVG**	**HEG**	**HVG**
**HECLIP**	**0.2177**	**0.2289**	**0.26**	**0.2033**	**0.227**	**0.2382**	**0.3449**	**0.1927**
**BLEEP**	0.1945	0.2155	0.227	0.1655	0.1553	0.1655	0.227	0.1423
**CLIP**	0.1761	0.1682	0.1763	0.1435	0.1277	0.1231	0.1005	0.1124
**HIist2ST**	0.1526	0.1231	0.1715	0.1043	0.1033	0.1097	0.0806	0.081
**HisToGene**	0.0497	0.0795	0.1368	0.0702	0.0906	0.0988	0.0411	0.0806
**ST-Net**	0.0881	0.0977	0.1427	0.75	0.0765	0.0897	0.0693	0.0791

Table S2: Experiment results of PCC (HEG and HVG introduced by BLEEP) on all datasets. HEG represents the average PCC of the 50 genes with the highly expression levels, and HVG represents the average PCC of the 50 highly variable genes.

Table S2 shows the experimental results of different methods on multiple datasets, analyzing the performance of HECLIP, BLEEP, CLIP, Hist2ST, HisToGene and ST-Net under HEG (Highly Expression Genes) and HVG (Highly Variable Genes). Among them, HECLIP performs very well on multiple datasets. On HEG of the GSE240429_HVG dataset, HECLIP reaches 0.2766, while the PCCs of other methods such as BLEEP, CLIP, Hist2ST, HisToGene and ST-Net are 0.1751, 0.1235, 0.0973, 0.0527 and 0.0983 respectively. On HEG of the GSE245620_HEG dataset, HECLIP reached 0.4297, which is significantly ahead of BLEEP's 0.4016, CLIP's 0.3134, Hist2ST's 0.2681, HisToGene's 0.2082, and ST-Net's 0.1763.

Table S3 shows the experimental results of HECLIP, BLEEP, CLIP, etc. on different datasets (such as GSE240429_HVG, GSE245620_HVG, etc.). The values in the table represent the median and mean error (RMSE) of the processing results of these methods on each dataset. The smaller the value, the better the performance of the method.

Table S4 shows the SSIM results of different methods on multiple datasets. The methods used include HECLIP, BLEEP, CLIP, Hist2ST, HisToGene, ST-Net, etc., and the datasets include GSE240429_HVG, GSE240429_HEG, GSE245620_HVG, etc. The values in the table represent the median and mean structural similarity index (SSIM) of these methods on different datasets. These data are used to evaluate the performance of each method on different datasets. Higher values of the SSIM index mean better results.

	GSE240429_HVG			GSE240429_HEG	
	Median	Mean		Median	Mean
HECLIP	**1.399610197**	**1.387534783**	HECLIP	**1.370712409**	**1.355543793**
BLEEP	1.430534139	1.422012842	BLEEP	1.413792867	1.407323134
CLIP	1.626552495	1.627038213	CLIP	1.594925955	1.595884287
Hist2ST	1.615386705	1.607585848	Hist2ST	1.563614816	1.557812068
HisToGene	1.769497517	1.770653183	HisToGene	1.699191251	1.700757467
ST-Net	1.80615625	1.807406305	ST-Net	1.70453805	1.706435812
	GSE245620_HVG			GSE245620_HEG	
	Median	Mean		Median	Mean
HECLIP	**1.389773662**	**1.374332369**	HECLIP	**1.36325393**	**1.34616768**
BLEEP	1.417242904	1.404545959	BLEEP	1.396270554	1.380206191
CLIP	1.614894806	1.615838656	CLIP	1.50463859	1.505206755
Hist2ST	1.537409561	1.524959818	Hist2ST	1.5112454	1.495579587
HisToGene	1.689162174	1.690761517	HisToGene	1.65844002	1.660737375
ST-Net	1.714713174	1.716474131	ST-Net	1.665884114	1.666630313
	spatialLIBD_1_HVG			spatialLIBD_1_HEG	
	Median	Mean		Median	Mean
HECLIP	**1.403946067**	**1.389519011**	HECLIP	**1.376833943**	**1.361987641**
BLEEP	1.43407773	1.421187506	BLEEP	1.408844157	1.397572964
CLIP	1.52463669	1.525318407	CLIP	1.504846201	1.505657861
Hist2ST	1.578286724	1.566584462	Hist2ST	1.541713676	1.5301147
HisToGene	1.588401434	1.590356132	HisToGene	1.569218763	1.570871337
ST-Net	1.595045552	1.596721686	ST-Net	1.654229999	1.65609765
	spatialLIBD_2_HVG			spatialLIBD_2_HEG	
	Median	Mean		Median	Mean
HECLIP	**1.404671196**	**1.39326937**	HECLIP	**1.357617931**	**1.342320741**
BLEEP	1.434753938	1.426232837	BLEEP	1.401604976	1.389772603
CLIP	1.524705489	1.525271889	CLIP	1.485359561	1.486138434
Hist2ST	1.553183247	1.546215685	Hist2ST	1.513306485	1.501640379
HisToGene	1.58844002	1.590736075	HisToGene	1.517693448	1.519445617
ST-Net	1.575884114	1.576567382	ST-Net	1.526750029	1.528981271

Table S3: Experimental results of mean and median RMSE.

	GSE240429_HVG			GSE240429_HEG	
	Median	Mean		Median	Mean
HECLIP	**0.007049112**	**0.018899016**	HECLIP	**0.023823292**	**0.045940236**
BLEEP	0.002460898	0.013528091	BLEEP	0.016050468	0.031158943
CLIP	-0.000925503	0.010077216	CLIP	0.007562715	0.022708358
Hist2ST	-0.004040602	0.006962407	Hist2ST	0.000986071	0.016111192
HisToGene	-0.006018241	0.005075103	HisToGene	-0.002474949	0.012705976
ST-Net	-0.004922625	0.006070427	ST-Net	0.000570276	0.01570182
	GSE245620_HVG			GSE245620_HEG	
	Median	Mean		Median	Mean
HECLIP	**0.011051967**	**0.027374213**	HECLIP	**0.025374469**	**0.044215903**
BLEEP	0.004406655	0.025296759	BLEEP	0.022622912	0.041710598
CLIP	-3.76E-05	0.020846101	CLIP	0.006243102	0.025260091
Hist2ST	-1.13E-03	0.019746472	Hist2ST	-0.003863461	0.015382363
HisToGene	-0.005003987	0.015843824	HisToGene	0.000151108	0.019258609
ST-Net	-0.006019055	0.014839508	ST-Net	-0.004735039	0.014253085
	spatialLIBD_1_HVG			spatialLIBD_1_HEG	
	Median	Mean		Median	Mean
HECLIP	**0.008800368**	**0.032249379**	HECLIP	**0.018775349**	**0.034528301**
BLEEP	-7.65E-05	0.038347337	BLEEP	0.015341608	0.028917029
CLIP	-0.01243205	0.025896798	CLIP	0.007873094	0.021466176
Hist2ST	-0.016532238	0.021795215	Hist2ST	0.0043134	0.017865025
HisToGene	-0.019426247	0.01889549	HisToGene	0.002844126	0.016464055
ST-Net	-0.020452476	0.01788954	ST-Net	0.004891875	0.01845925
	spatialLIBD_2_HVG			spatialLIBD_2_HEG	
	Median	Mean		Median	Mean
HECLIP	**0.008607939**	**0.030900096**	HECLIP	**0.028549378**	**0.047821485**
BLEEP	0.000481062	0.034169565	BLEEP	0.021034269	0.03591831
CLIP	-0.004897877	0.028719041	CLIP	0.0144811	0.029467748
Hist2ST	-0.007052268	0.026626773	Hist2ST	0.011948241	0.026866023
HisToGene	-0.008955454	0.024717654	HisToGene	0.010517467	0.025465374
ST-Net	-0.009978791	0.023712058	ST-Net	0.011553265	0.026460068

Table S4: Experimental results of mean and median SSIM.

	**Mouse Brain Serial Section 2 _HVG**					**Mouse Brain Serial Section 2 _HEG**				
	**Hit@5**	**Hit@4**	**Hit@3**	**Hit@2**	**Hit@1**	**Hit@5**	**Hit@4**	**Hit@3**	**Hit@2**	**Hit@1**
**HECLIP**	**0.9919**	**0.9858**	**0.9595**	**0.8654**	**0.5395**	**1**	**0.998**	**0.9919**	**0.9747**	**0.8451**
**BLEEP**	0.9903	0.9822	0.9378	0.8213	0.4345	0.9972	0.9912	0.9608	0.9137	0.6788
	**Human Brain Cancer_HVG**					**Human Brain Cancer_HEG**				
	**Hit@5**	**Hit@4**	**Hit@3**	**Hit@2**	**Hit@1**	**Hit@5**	**Hit@4**	**Hit@3**	**Hit@2**	**Hit@1**
**HECLIP**	**1**	**1**	**1**	**0.9929**	**0.9988**	**1**	**1**	**1**	0.998	0.9879
**BLEEP**	1	1	0.9985	0.9918	0.9769	1	1	1	**1**	**0.9991**
	**Xenium_FFPE_Human_Breast_Cancer_HVG**					**Xenium_FFPE_Human_Breast_Cancer_HEG**				
	**Hit@5**	**Hit@4**	**Hit@3**	**Hit@2**	**Hit@1**	**Hit@5**	**Hit@4**	**Hit@3**	**Hit@2**	**Hit@1**
**HECLIP**	**0.9527**	**0.9112**	**0.8651**	**0.7288**	**0.5094**	**0.9332**	**0.8992**	**0.8446**	**0.7245**	**0.5218**
**BLEEP**	0.8907	0.8459	0.756	0.6237	0.4997	0.9068	0.865	0.7905	0.645	0.3613

Table S5: Experimental results of Hit@T on the dataset Human Brain Cancer, Mouse Brain Serial Section 2 and Xenium FFPE Human Breast Cancer.

Table S5 shows the Hit@T performance of the HECLIP and BLEEP models on the Human Brain Cancer and Mouse Brain Serial Section 2 datasets. For example, for the Mouse Brain Serial Section 2 HVG dataset, under the Hit@1 metric, HECLIP scores 0.5395, while BLEEP scores only 0.4345. This shows that in the first prediction result, HECLIP has a significantly higher proportion of correct identifications than BLEEP. Under the Hit@2 metric, HECLIP scores 0.8654, while BLEEP scores 0.8213. Similarly, HECLIP also has a higher proportion of correct predictions in the first two prediction results than BLEEP. Overall, the HECLIP model outperforms BLEEP in multiple datasets and metrics, especially for the Hit@1 metric for the Mouse Brain Serial Section 2_HVG dataset and the Hit@2 metric for the Human Brain Cancer_HEG dataset. HECLIP significantly outperforms the comparison method BLEEP on both Xenium_FFPE_Human_Breast_Cancer_HVG and Xenium_FFPE_Human_Breast_Cancer_HEG. On the HVG dataset, HECLIP achieved 0.5094, 0.7288, 0.8651, 0.9112, and 0.9527 on the Hit@1 to Hit@5 indicators, which are ahead of the corresponding scores of BLEEP (the highest is only 0.8907). On the HEG dataset, HECLIP also performed well, with Hit@1 to Hit@5 scores of 0.5218, 0.7245, 0.8446, 0.8992, and 0.9332, which are significantly improved compared to BLEEP's 0.3613 to 0.9068.

	**Mouse Brain Serial Section 2 _HVG**		**Mouse Brain Serial Section 2 _HEG**	
	**HEG**	**HVG**	**HEG**	**HVG**
**HECLIP**	**0.6764**	**0.7261**	**0.6995**	**0.7178**
**BLEEP**	0.4672	0.51	0.6162	0.633
	**Human Brain Cancer_HVG**		**Human Brain Cancer_HEG**	
	**HEG**	**HVG**	**HEG**	**HVG**
**HECLIP**	**0.7321**	**0.825**	**0.7887**	**0.8076**
**BLEEP**	0.5188	0.5798	0.6945	0.6773
	**Xenium_FFPE_Human_Breast_Cancer_HVG**		**Xenium_FFPE_Human_Breast_Cancer_HEG**	
	**HEG**	**HVG**	**HEG**	**HVG**
**HECLIP**	**0.5972**	**0.5828**	**0.5441**	**0.5705**
**BLEEP**	0.4696	0.454	0.4769	0.4387

Table S6: Experimental results of PCC on the dataset Human Brain Cancer and Mouse Brain Serial Section 2. HEG represents the average PCC of the 50 genes with the highly expression levels, and HVG represents the average PCC of the 50 highly variable genes.

Table S6 shows the PCC (Pearson correlation coefficient) results obtained using the HECLIP and BLEEP methods on the "Human Brain Cancer" and "Mouse Brain Serial Section 2" datasets. In Mouse Brain Serial Section 2_HVG, the PCC values of the HECLIP method are 0.6764 and 0.7261, which are better than the 0.4672 and 0.51 of the BLEEP method. In Human Brain Cancer_HEG, the PCC values of the HECLIP method are 0.7887 and 0.8076, while the PCC values of the BLEEP method are only 0.6945 and 0.6773. Overall, HECLIP performs better than BLEEP in different datasets and gene types. On the Xenium_FFPE_Human_Breast_Cancer dataset, HECLIP showed superior performance in both highly expressed genes (HEG) and highly variable genes (HVG). On the HVG subset, HECLIP achieved average correlation scores of 0.5828 and 0.5705 in the two data indicators, significantly better than BLEEP's 0.454 and 0.4387. On the HEG feature set, HECLIP also achieved high scores of 0.5972 and 0.5441, ahead of BLEEP's 0.4696 and 0.4769. These results show that HECLIP can not only model the spatial structure more accurately on highly expressed genes, but also has stronger modeling and alignment capabilities at the level of highly variable genes, fully verifying its robustness and advantages in cross-modal gene expression prediction.

B Ablation study

RMSE ↓	Median	Mean	Median	Mean
	**GSE240429_HVG**		**GSE240429_HEG**	
**HECLIP**	**1.3996**	**1.3875**	**1.3707**	**1.3555**
**w/o loss**	1.4071	1.3973	1.3809	1.3629
**w/o data**	1.4104	1.3985	1.3882	1.3656
	**GSE245620_HVG**		**GSE245620_HEG**	
**HECLIP**	**1.3898**	**1.3743**	**1.3632**	**1.3461**
**w/o loss**	1.3967	1.3839	1.3778	1.3555
**w/o data**	1.3989	1.38441	1.3767	1.3522
	**spatialLIBD_1_HVG**		**spatialLIBD_1_HEG**	
**HECLIP**	**1.4039**	**1.3895**	**1.3768**	**1.3619**
**w/o loss**	1.4171	1.3998	1.3838	1.3729
**w/o data**	1.4209	1.4005	1.3891	1.3733
	**spatialLIBD_2_HVG**		**spatialLIBD_2_HEG**	
**HECLIP**	**1.4047**	**1.3932**	**1.3576**	**1.3423**
**w/o loss**	1.4118	1.4028	1.3617	1.3532
**w/o data**	1.4201	1.4047	1.3622	1.3554

Table S7: Ablation experiment results of RMSE on all datasets. The downward arrow means lower is better.

Table S7 shows the results of ablation experiments on different datasets, and the model performance is evaluated by the root mean square error (RMSE). HECLIP represents the original complete HECLIP model, which is our baseline model. w/o loss is the HECLIP model after removing a specific loss function. w/o data is the HECLIP model after removing a part of the data. The lower the RMSE value, the better the model effect, so the downward arrow in the table indicates that the lower RMSE value is a better result. For the GSE204429_HVG dataset, the Median RMSE of the HECLIP model is 1.3996 and the Mean RMSE is 1.3875. After removing the image-centric loss function (w/o loss), the Median RMSE is 1.4071 and the Mean RMSE is 1.3973. After removing data augmentation (w/o data), the Median RMSE is 1.4104 and the Mean RMSE is 1.3985. In the SpatialLIBD_2_HEG dataset, the Median RMSE of the HECLIP model is 1.3576 and the Mean RMSE is 1.3423. After removing the image-centric loss function (w/o loss), the Median RMSE is 1.4188 and the Mean RMSE is 1.3532. After removing data augmentation (w/o data), the Median RMSE is 1.4201 and the Mean RMSE is 1.3554. The ablation experiment results in Table 5 show that the RMSE values of the complete HECLIP model on different datasets are generally lower than those of the model after removing the image-centric loss function or data augmentation, demonstrating the effectiveness of its components and the superiority of its overall performance. These results further verify the rationality of the construction of the HECLIP model and the important role of each component in improving model performance.

	**GSE240429_HVG**		**GSE240429_HEG**		**GSE245620_HVG**		**GSE245620_HEG**	
	**HEG**	**HVG**	**HEG**	**HVG**	**HEG**	**HVG**	**HEG**	**HVG**
**HECLIP**	**0.2766**	**0.2124**	**0.255**	**0.1933**	**0.2753**	**0.263**	**0.4297**	**0.2289**
**w/o loss**	0.2556	0.204	0.234	0.1761	0.2194	0.2272	0.3993	0.1983
**w/o data**	0.2681	0.2072	0.2411	0.1853	0.2632	0.2518	0.4107	0.2123
	**spatialLIBD_1_HVG**		**spatialLIBD_1_HEG**		**spatialLIBD_2_HVG**		**spatialLIBD_2_HEG**	
	**HEG**	**HVG**	**HEG**	**HVG**	**HEG**	**HVG**	**HEG**	**HVG**
**HECLIP**	**0.2177**	**0.2289**	**0.26**	**0.2033**	**0.227**	**0.2382**	**0.3449**	**0.1927**
**w/o loss**	0.2	0.2113	0.2547	0.1989	0.1857	0.2133	0.3323	0.1871
**w/o data**	0.1988	0.2097	0.2563	0.2002	0.2018	0.2357	0.3319	0.1922

Table S8: Ablation experiment results of PCC on all datasets. HEG represents the average PCC of the 50 genes with the highly expression levels, and HVG represents the average PCC of the 50 highly variable genes.

Table S8 shows the ablation results of the HECLIP method on PCC. The ablation experiment includes the effects of removing the loss function (w/o loss) and removing data augmentation (w/o data)

on the model performance. On the GSE245620_HEG dataset, HECLIP's HEG value reaches 0.4297, compared with the PCC values of w/o loss and w/o data of 0.3993 and 0.4107, respectively. On the SpatialLIBD_2_HEG dataset, HECLIP's HEG value is 0.3449, while without loss function (w/o loss) and without data augmentation (w/o data), its PCC value drops to 0.3233 and 0.3319, respectively. Although these two simplified versions still show some effect, in comparison, the performance of our complete HECLIP model is the most superior. This proves the effectiveness of our method.

	**GSE240429_HVG**		**GSE240429_HEG**		**GSE245620_HVG**		**GSE245620_HEG**	
	**HEG**	**HVG**	**HEG**	**HVG**	**HEG**	**HVG**	**HEG**	**HVG**
**HECLIP**	**0.2766**	**0.2124**	**0.255**	**0.1933**	**0.2753**	**0.263**	**0.4297**	**0.2289**
**w/pair**	0.0691	0.1853	0.2131	0.1621	0.2138	0.1943	0.3957	0.2047
	**spatialLIBD_1_HVG**		**spatialLIBD_1_HEG**		**spatialLIBD_2_HVG**		**spatialLIBD_2_HEG**	
	**HEG**	**HVG**	**HEG**	**HVG**	**HEG**	**HVG**	**HEG**	**HVG**
**HECLIP**	**0.2177**	**0.2289**	**0.26**	**0.2033**	**0.227**	**0.2382**	**0.3449**	**0.1927**
**w/pair**	0.1554	0.1328	0.2315	0.1599	0.0987	0.2112	0.3306	0.1762

Table S9: Experimental results of paired data augmentation on PCC. HEG represents the average PCC of the 50 genes with the highly expression levels, and HVG represents the average PCC of the 50 highly variable genes.

Table S9 shows the experimental results of paired data augmentation on PCC. The HVG value without paired data augmentation on GSE240429_HVG is 0.2124, and the value with paired data augmentation (w/pair) is 0.1853. The HEG value without paired data augmentation on GSE245620_HEG is 0.4297, and the PCC value with paired data augmentation (w/pair) is 0.3957. This shows that the PCC value after paired data augmentation is lower than the PCC value without paired data augmentation, indicating that paired data augmentation does not improve the model performance of this particular dataset and method combination.

			**GSE240429_HVG**					**GSE240429_HEG**		
	**Hit@5**	**Hit@4**	**Hit@3**	**Hit@2**	**Hit@1**	**Hit@5**	**Hit@4**	**Hit@3**	**Hit@2**	**Hit@1**
**HECLIP**	**0.9982**	**0.9952**	**0.9873**	**0.9627**	**0.8208**	**1**	**1**	**1**	**1**	**0.9978**
**w/pair**	0.9965	0.9903	0.964	0.9416	0.8182	**1**	**1**	**1**	**1**	**0.9978**
			**GSE245620_HVG**					**GSE245620_HEG**		
	**Hit@5**	**Hit@4**	**Hit@3**	**Hit@2**	**Hit@1**	**Hit@5**	**Hit@4**	**Hit@3**	**Hit@2**	**Hit@1**
**HECLIP**	**0.9978**	**0.9968**	**0.9942**	**0.9772**	**0.8634**	**0.9998**	**0.9998**	**0.9998**	**0.999**	**0.9926**
**w/pair**	0.9874	0.9806	0.9706	0.9471	0.8496	**0.9998**	**0.9998**	**0.9998**	**0.999**	**0.9926**
			**spatialLIBD_1_HVG**					**spatialLIBD_1_HEG**		
	**Hit@5**	**Hit@4**	**Hit@3**	**Hit@2**	**Hit@1**	**Hit@5**	**Hit@4**	**Hit@3**	**Hit@2**	**Hit@1**
**HECLIP**	**0.9712**	**0.9463**	**0.8839**	**0.688**	**0.3688**	**1**	**0.9998**	**0.9994**	**0.9793**	**0.5999**
**w/pair**	0.9647	0.93	0.8572	0.6816	0.3341	1	0.9992	0.9977	0.9697	0.5897
			**spatialLIBD_2_HVG**					**spatialLIBD_2_HEG**		
	**Hit@5**	**Hit@4**	**Hit@3**	**Hit@2**	**Hit@1**	**Hit@5**	**Hit@4**	**Hit@3**	**Hit@2**	**Hit@1**
**HECLIP**	**1**	**0.9996**	**0.9987**	**0.976**	**0.5968**	**1**	**1**	0.9993	**0.9917**	0.6214
**w/pair**	0.9791	0.944	0.856	0.629	0.238	1	1	**0.9995**	0.9905	**0.6302**

Table S10: Experimental results of paired data augmentation on Hit@T.

Table S10 shows the experimental results of paired data augmentation on four different datasets, evaluating the Hit@T performance of the HECLIP model with and without paired data augmentation. The Hit@T metric indicates the proportion of the correct answer in the first T prediction results, and there are five cases: Hit@5, Hit@4, Hit@3, Hit@2, and Hit@1. For the GSE204429_HVG dataset, the HECLIP model scored 0.9627 under the Hit@2 metric and 0.8208 under the Hit@1 metric. The model with paired data augmentation (w/pair) scored 0.9416 under the Hit@2 metric and 0.8182 under the Hit@1 metric. By comparison, the original HECLIP model performs better under these metrics. For the GSE245620_HEG dataset, the HECLIP model scored 0.9998 under the Hit@5 metric and 0.9998 under the Hit@3 metric. The model with paired data augmentation (w/pair) also scores 0.9998 under

the Hit@5 metric and 0.9998 under the Hit@3 metric. In this dataset, the performance under the two conditions is almost the same, but the HECLIP model is slightly ahead in some evaluation indicators. In summary, the ablation experiment results in Table 8 show that the original HECLIP model performs better than the model with paired data augmentation (w/pair) on some datasets and indicators.

RMSE	GSE240429_HVG		GSE240429_HEG	
	Mean	Median	Mean	Median
HECLIP	**1.399610197**	**1.387534783**	**1.370712409**	**1.355543793**
w/pair	1.494453172	1.474690092	1.408808703	1.397724193
	spatialLIBD_1_HVG		spatialLIBD_1_HEG	
HECLIP	**1.403946067**	**1.389519011**	**1.376833943**	**1.361987641**
w/pair	1.412601848	1.404350247	1.388164227	1.379384432
	GSE245620_HVG		GSE245620_HEG	
HECLIP	**1.389773662**	**1.374332369**	**1.36325393**	**1.34616768**
w/pair	1.399270708	1.384572017	1.39216724	1.381982154
	spatialLIBD_2_HVG		spatialLIBD_2_HEG	
HECLIP	**1.404671196**	**1.39326937**	**1.357617931**	**1.342320741**
w/pair	1.411937733	1.400743227	1.379904923	1.363448507
SSIM	GSE240429_HVG		GSE240429_HEG	
	Mean	Median	Mean	Median
HECLIP	**0.007049112**	**0.018899016**	**0.023823292**	**0.045940236**
w/pair	0.058768452	0.013271053	0.016810014	0.028316614
	spatialLIBD_1_HVG		spatialLIBD_1_HEG	
HECLIP	**0.008800368**	**0.032249379**	**0.018775349**	**0.034528301**
w/pair	0.00685557	0.021008097	0.01136385	0.021008097
	GSE245620_HVG		GSE245620_HEG	
HECLIP	**0.011051967**	**0.027374213**	**0.025374469**	**0.044215903**
w/pair	0.007863162	0.01512229	0.018707264	0.038989104
	spatialLIBD_2_HVG		spatialLIBD_2_HEG	
HECLIP	**0.008607939**	**0.030900096**	**0.028549378**	**0.047821485**
w/pair	0.006443838	0.022378296	0.0189363	0.03996825

Table S11: Experimental results of paired data augmentation on RMSE and SSIM.

Table S11 shows the RMSE (root mean square error) and SSIM (structural similarity index) results on multiple datasets (GSE240429, GSE245620, spatialLIBD_1, spatialLIBD_2), comparing HECLIP and paired data augmentation (w/pair) methods. Lower RMSE and higher SSIM indicate better model performance.

From the data, we can see that HECLIP's RMSE is significantly lower than paired data augmentation methods, and SSIM is also better, indicating that HECLIP has smaller prediction errors and higher generation quality, while paired data augmentation reduces model performance. For example, for the GSE240429_HVG dataset, HECLIP's RMSE is 1.3996 and 1.3875, while paired data augmentation's RMSE is 1.4945 and 1.4746, indicating that paired data augmentation leads to a significant increase in RMSE and an increase in error. HECLIP's SSIM is 0.0070 and 0.0189, and paired data augmentation's SSIM is 0.0587 and 0.0132. SSIM decreases instead, indicating that paired data augmentation does not improve the image structure similarity. For the spatialLIBD_1_HVG dataset, HECLIP's RMSE is 1.4039 and 1.3895, and paired data augmentation's RMSE is 1.4126 and 1.4044. HECLIP's SSIM is 0.0088 and 0.0322, and paired data augmentation's SSIM is 0.0069 and 0.0210. The structural similarity decreases, indicating that paired data augmentation has a negative impact on the quality of the results.

HECLIP's RMSE on all datasets is significantly lower than that of paired data augmentation methods, indicating that its predictions are more accurate. At the same time, HECLIP's SSIM is generally higher, indicating that the generated results are of better quality. On the contrary, the paired data augmentation method not only failed to reduce RMSE, but also led to an increase in error and a decrease in SSIM, indicating that it did not bring the expected improvement in the current experiment.

C Hyperparameter study

	RMSE↓		SSIM↑		RMSE↓		SSIM↑	
	**Median**	**Mean**	**Median**	**Mean**	**Median**	**Mean**	**Median**	**Mean**
	**GSE240429_HVG**				**GSE240429_HEG**			
**TOP10**	1.4106	1.399	0.007967	0.013892	1.3933	1.3834	**0.02412**	0.04246
**TOP30**	1.4128	1.4015	**0.018361**	**0.044911**	1.3848	1.3713	0.02275	0.0442
**TOP50**	**1.3996**	**1.3875**	0.007049	0.018899	1.3707	1.3555	0.02382	**0.0459**
**TOP100**	1.4075	1.3955	0.003771	0.013978	1.3728	1.3576	0.02213	0.04446
**TOP200**	1.405	1.3937	0.002716	0.012189	**1.3667**	**1.3519**	0.02158	0.04309
	**GSE245620_HVG**				**GSE245620_HEG**			
**TOP10**	1.407	1.394	**0.03113**	**0.07443**	1.3912	1.3789	**0.02756**	0.04281
**TOP30**	1.4023	1.3876	0.01343	0.03583	1.3791	1.3634	0.02469	0.04276
**TOP50**	**1.3897**	**1.3743**	0.01105	0.02737	1.3632	1.3461	0.02531	0.04421
**TOP100**	1.3956	1.3802	0.006	0.0179	1.3643	1.3466	0.02493	0.04404
**TOP200**	1.3913	1.3764	0.00414	0.01447	**1.3555**	**1.3374**	0.02607	**0.04478**
	**spatialLIBD_1_HVG**				**spatialLIBD_1_HEG**			
**TOP10**	1.4147	1.4033	**0.04218**	0.1051	1.3987	1.3876	**0.02088**	0.03427
**TOP30**	1.4145	1.4009	0.01328	**0.04828**	1.3908	1.3771	0.01847	0.03359
**TOP50**	**1.4039**	**1.3895**	0.0088	0.03224	1.3768	1.3619	0.01877	**0.03452**
**TOP100**	1.4124	1.3981	0.00374	0.01681	1.3812	1.3663	0.01727	0.03273
**TOP200**	1.4111	1.3971	0.00192	0.01029	**1.3756**	**1.3613**	0.01681	0.0317
	**spatialLIBD_2_HVG**				**spatialLIBD_2_HEG**			
**TOP10**	1.4152	1.4072	**0.03992**	**0.0901**	1.3876	1.375	**0.02951**	0.04597
**TOP30**	1.4151	1.4043	0.01331	0.0434	1.3747	1.3594	0.02753	0.04659
**TOP50**	**1.4046**	**1.3932**	0.0086	0.0309	1.3576	1.3423	0.02854	**0.04782**
**TOP100**	1.414	1.4016	0.00412	0.0175	1.3599	1.3443	0.02717	0.04638
**TOP200**	1.4129	1.4009	0.00242	0.011	**1.3524**	**1.3383**	0.02636	0.04513

Table S12: Top K hyperparameter selection experiments on RMSE and SSIM. TOP50 is the default parameter used for results reported in papers.

We conducted a comprehensive hyperparameter study to assess the impact of varying the Top-K feature selection parameter on model performance across multiple datasets. As shown in Table S13, increasing K generally improves performance in terms of both RMSE and SSIM up to a certain point. For highly variable genes (HVG), K = 50 consistently achieves a favorable balance, yielding low RMSE and high SSIM across datasets such as GSE240429, GSE245620, and spatialLIBD 1 and 2. For example, on the GSE240429 HVG dataset, the mean RMSE drops to 1.3875 and SSIM improves to 0.0189 at K = 50. Similarly, in the HEG setting, performance continues to improve up to K = 200, but gains diminish beyond that, indicating a plateau. These findings support the use of K = 50 as the default setting in our main experiments.

Further evaluation using Pearson correlation coefficient (PCC) and Hit@T metrics, shown in Tables S13 and S14, reinforces these trends. PCC improves substantially when increasing K from 10 to 50 across all datasets. For instance, in the GSE240429 HVG dataset, PCC increases from 0.1671 (K=10) to 0.2124 (K=50), and similar improvements are observed across other datasets. However, beyond K = 200, PCC gains diminish or slightly reverse. The Hit@T metrics show high and stable performance across values of K, with minimal variation, indicating that Top-K selection has less influence on top-ranked gene predictions. Overall, these results demonstrate that our defaulting setting of K =50 maintains a balance between accuracy and generalizability of prediction results.

	GSE240429_HVG		GSE240429_HEG		GSE245620_HVG		GSE245620_HEG	
	**HEG**	**HVG**	**HEG**	**HVG**	**HEG**	**HVG**	**HEG**	**HVG**
**TOP10**	0.2376	0.1671	0.2151	0.1457	0.2339	0.2189	0.3421	0.165
**TOP30**	0.2698	0.2026	0.2473	0.1761	0.2674	0.2539	0.4081	0.209
**TOP50**	0.2766	0.2124	**0.255**	0.1933	0.2753	0.263	0.4297	0.2289
**TOP100**	**0.2819**	0.2275	0.2458	0.2024	0.2848	0.2734	0.4491	0.2452
**TOP200**	0.2778	**0.2334**	0.247	**0.2085**	**0.2894**	**0.2785**	**0.4703**	**0.2633**
**TOP1000**	0.2389	0.2071	0.2311	0.2144	0.27	0.2574	0.4703	0.2607
	**spatialLIBD_1_HVG**		**spatialLIBD_1_HEG**		**spatialLIBD_2_HVG**		**spatialLIBD_2_HEG**	
	**HEG**	**HVG**	**HEG**	**HVG**	**HEG**	**HVG**	**HEG**	**HVG**
**TOP10**	0.1836	0.2049	0.2173	0.1655	0.1539	0.1931	0.3058	0.15
**TOP30**	0.2109	0.2324	0.2479	0.1942	0.1859	0.2277	0.3352	0.1803
**TOP50**	0.2177	0.2289	0.26	0.2033	**0.227**	0.2382	0.3449	0.1927
**TOP100**	**0.2245**	**0.2465**	0.2689	0.2141	0.2039	**0.2484**	0.3539	0.2068
**TOP200**	0.2241	**0.2465**	**0.2738**	**0.2206**	0.2019	0.2468	**0.3569**	**0.2131**
**TOP1000**	0.1977	0.2173	0.2669	0.2193	0.168	0.2093	0.3503	0.2077

Table S13: Top K hyperparameter selection experiments on PCC. K=50 is the default parameter used for results reported in papers. HEG represents the average PCC of the 50 genes with the highly expression levels, and HVG represents the average PCC of the 50 highly variable genes.

	GSE240429_HVG					GSE240429_HEG				
	**Hit@5**	**Hit@4**	**Hit@3**	**Hit@2**	**Hit@1**	**Hit@5**	**Hit@4**	**Hit@3**	**Hit@2**	**Hit@1**
**top10**	0.9965	0.9943	0.9864	0.9609	0.8103	**1**	**1**	**1**	**1**	0.9974
**top30**	0.9978	**0.996**	**0.9873**	**0.9635**	0.8199	**1**	**1**	**1**	**1**	**0.9978**
**top50**	0.9982	0.9952	**0.9873**	0.9627	0.8208	**1**	**1**	**1**	**1**	**0.9978**
**top100**	0.9982	0.9947	0.9859	0.9631	0.8199	**1**	**1**	**1**	**1**	**0.9978**
**top200**	**0.9987**	0.9943	0.9855	0.9605	**0.823**	**1**	**1**	**1**	**1**	**0.9978**
	**GSE245620_HVG**					**GSE245620_HEG**				
	**Hit@5**	**Hit@4**	**Hit@3**	**Hit@2**	**Hit@1**	**Hit@5**	**Hit@4**	**Hit@3**	**Hit@2**	**Hit@1**
**top10**	0.9974	0.995	0.9912	0.9655	0.8438	**0.9998**	**0.9998**	**0.9998**	0.9988	0.992
**top30**	0.9972	0.996	0.9934	0.973	0.857	**0.9998**	**0.9998**	**0.9998**	**0.999**	**0.9926**
**top50**	0.9978	0.9968	0.9942	0.9772	0.8634	**0.9998**	**0.9998**	**0.9998**	**0.999**	**0.9926**
**top100**	0.998	0.9968	0.9948	0.9776	**0.867**	**0.9998**	**0.9998**	**0.9998**	**0.999**	**0.9926**
**top200**	**0.9984**	**0.997**	**0.9952**	**0.9788**	0.8652	**0.9998**	**0.9998**	**0.9998**	**0.999**	**0.9926**
	**spatialLIBD_1_HVG**					**spatialLIBD_1_HEG**				
	**Hit@5**	**Hit@4**	**Hit@3**	**Hit@2**	**Hit@1**	**Hit@5**	**Hit@4**	**Hit@3**	**Hit@2**	**Hit@1**
**top10**	0.9639	0.9353	0.8664	0.6753	0.3589	**1**	**1**	0.9992	0.9787	0.5707
**top30**	0.9706	0.9445	0.8801	0.6914	0.3612	**1**	0.9998	0.9992	0.9791	0.6035
**top50**	0.9712	0.9463	**0.8839**	0.688	0.3688	**1**	0.9998	0.9994	0.9793	0.5999
**top100**	**0.9724**	0.9447	0.8829	0.6926	0.3717	**1**	0.9998	0.9994	**0.9812**	**0.6039**
**top200**	0.9697	**0.9472**	0.8833	**0.6953**	**0.3786**	**1**	0.9998	**0.999**	0.9787	0.5907
	**spatialLIBD_2_HVG**					**spatialLIBD_2_HEG**				
	**Hit@5**	**Hit@4**	**Hit@3**	**Hit@2**	**Hit@1**	**Hit@5**	**Hit@4**	**Hit@3**	**Hit@2**	**Hit@1**
**top10**	**1**	0.9979	0.9852	0.9682	0.5993	**1**	**1**	**0.9998**	0.9891	0.6119
**top30**	**1**	0.9974	0.9966	0.9695	0.6073	**1**	**1**	0.9993	0.991	0.618
**top50**	**1**	**0.9996**	**0.9987**	**0.976**	0.5968	**1**	**1**	0.9993	0.9917	0.6214
**top100**	**1**	0.9972	0.9958	0.9712	**0.6195**	**1**	**1**	0.9995	0.9915	0.626
**top200**	**1**	0.9964	0.9951	0.9702	0.6051	**1**	**1**	0.9995	**0.9922**	**0.6331**

Table S14: Top K hyperparameter selection experiments on Hit@T. TOP50 is the default parameter used for results reported in papers.

D Cross dataset analysis

	**GSE240429_HVG->GSE245620_HVG**				
	**Hit@5**	**Hit@4**	**Hit@3**	**Hit@2**	**Hit@1**
**HECLIP**	**0.997**	**0.9683**	**0.885**	**0.2572**	0.0046
**BLEEP**	0.9871	0.9518	0.7677	0.2011	**0.0102**
**Hist2ST**	0.9779	0.8871	0.5698	0.1087	0.0008
	**GSE240429_HEG->GSE245620_HEG**				
	**Hit@5**	**Hit@4**	**Hit@3**	**Hit@2**	**Hit@1**
**HECLIP**	**0.9982**	**0.977**	**0.8966**	**0.3171**	**0.076**
**BLEEP**	0.9891	0.9677	0.8108	0.2333	0.0274
**Hist2ST**	0.9763	0.8796	0.6319	0.1147	0.0033

Table S15: Results of the cross-dataset prediction experiment on the Hit@T.

Table S15 shows the performance of three models, HECLIP, BLEEP and Hist2ST, in cross-dataset prediction experiments, evaluated by the Hit@T metric. Hit@T indicates the proportion of correct prediction results found in the Top T results, with T=5, 4, 3, 2, and 1. In the experiment of GSE204429_HVG $->$ GSE245620_HVG, HECLIP scored 0.997 in Hit@5, BLEEP scored 0.9871, and Hist2ST scored 0.9779. This shows that HECLIP performs best on this task. HECLIP scored 0.2572 in Hit@2, BLEEP scored 0.2011, and Hist2ST scored 0.1087. Similarly, HECLIP scored the highest. In the experiment of GSE204429_HEG $->$ GSE245620_HEG, HECLIP scored 0.8966 in Hit@3, while BLEEP scored 0.8108 and Hist2ST scored 0.6319, which again showed the advantage of HECLIP.

Table S16 shows the average RMSE (root mean square error) and SSIM (structural similarity index) results of the cross-dataset prediction experiment, comparing the three methods of HECLIP, BLEEP and Hist2ST. Lower RMSE and higher SSIM indicate better model performance. From the data, it can be seen that HECLIP has achieved the best performance in all experimental settings, with the lowest RMSE and the highest SSIM. For example, on GSE240429_HVG $\rightarrow$ GSE245620_HVG, HECLIP's RMSE is 1.873 and SSIM is 0.00692. In contrast, BLEEP's RMSE is 1.965 (higher), SSIM is 0.00333 (lower), Hist2ST's RMSE is 2.187 (higher), and SSIM is 0.00105 (lower). This shows that the results generated by HECLIP are closer to the target data, with smaller prediction errors and higher structural similarity.

In summary, these cross-dataset experiments demonstrate that HECLIP is not only effective under standard conditions but also robust in scenarios involving suboptimal reference data. Its reliance on local patch-based representations, rather than a strict dependence on global spatial context or dataset-specific features, likely contributes to this robustness. This design makes HECLIP especially well-suited for real-world applications, where well-matched reference datasets may be limited, unavailable, or of varying quality.

	**GSE240429_HVG->GSE245620_HVG**		**GSE240429_HEG->GSE245620_HEG**	
	**SSIM**	**RMSE**	**SSIM**	**RMSE**
**HECLIP**	**0.00692**	**1.873**	**0.00587**	**1.862**
**BLEEP**	0.00333	1.965	0.00388	1.973
**Hist2ST**	0.00105	2.187	0.00097	2.281

Table S16: Results of the cross-dataset prediction experiment on the average RMSE and SSIM.

E Summary of conventional methods

Model	Local Features	Global Features (spot-spatial relations)	Global Features (spot-neighborhood relations)	Global Features (multiple latent relations)	Parameters
DeepSpaCE [1]	VGG16	NA	Super Resolution	NA	137M
ST-Net [2]	Pretrained DenseNet 121	NA	NA	NA	8M
HisToGene [3]	ViT	NA	Super Resolution	NA	222M
DeepPT [4]	Pretrained ResNet50 + Autoencoder + MLP	NA	NA	NA	26M
Hist2ST [5]	ConvMixer	Transformer	GNN	NA	230M
THItoGene [6]	Omni-dimensional Dynamic Convolution + Efficient-CapsNet	Transformer	Graph Attention	NA	64M
CLIP [7]	ViT + Transformer + Contrastive Learning	NA	NA	NA	149M
BLEEP [8]	ViT + Contrastive Learning	NA	NA	NA	25M
HGGEP [9]	Gradient Enhancement + ShuffleNet V2	CBAM + Transformer	Hypergraph Association	Hypergraph Association + LSTM	116M
HECLIP (Ours)	ResNet50 + Data augmentation + image-centric loss	NA	NA	NA	55M

Table S17: Summary of conventional methods.

Table S17 summarizes a variety of common methods for this task and compares their required features, feature extraction methods, and trainable parameter amounts. Generally speaking, the information provided by HE images for whole slices includes global and local information. Global features include, for example, spot-spatial relations, spot-neighborhood relations, multiple latent relations, etc., while local features are only information within a single patch and spot. Different methods have different choices in extracting local and global features. For example, DeepSpaCE uses VGG16 for local feature extraction, while CLIP and BLEEP use ViT combined with Transformer for feature learning. Our method HECLIP uses ResNet50 + data enhancement + variant contrastive learning for feature extraction. The design is simple and efficient, avoiding complex global feature modeling while ensuring good performance. In addition, the trainable parameter amount of HECLIP is only 55M, which greatly reduces the computational cost compared to traditional models such as Hist2ST (230M) and CLIP (149M), making it easier to train and deploy.

The limitations of current prediction methods for gene expression from pathology images include: (1) Complex Model Structures: Many existing models have intricate architectures, making them computationally expensive and challenging to interpret. (2) Cumbersome Preprocessing: These methods often require extensive preprocessing steps, such as feature extraction or alignment, which increases complexity and may introduce biases. (3) Dependence on Global Information: Many models rely on global spatial relationships (e.g., relative spot coordinates) to infer gene expression, which may limit their applicability to cases where such global context is unavailable or unreliable. (4) Limited Adaptability: Due to their reliance on global context, these models may struggle to generalize across different datasets or imaging conditions. HECLIP, in contrast, simplifies the process by predicting gene expression using only local patch information, reducing preprocessing efforts and eliminating the dependency on global spatial relationships.

F Division of the training and testing datasets

- GSE240429 includes a total of 4 whole-slide H&E images, GEX_C73_A1_Merged, GEX_C73_B1_ Merged, GEX_C73_D1_Merged, and their respective spots were used as the training set, while GEX_C73_C1_ Merged and its respective spots were used as the test set.
- GSE245620 includes a total of 4 whole-slide H&E images, GSM7845914_GEX_PSC011-4_A1_Merged, GSM7845915_GEX_PSC011-4_B1_Merged, GSM7845917_GEX_PSC011-4_D1_Merged, and their respective spots were used as the training set, while GSM7845916_GEX_PSC011-4_C1_Merged and its respective spots were used as the test set.
- SpatialLIBD_1 includes a total of 4 whole-slide H&E images, 151507_full_image, 151508_full_image, 151510_full_image, and their respective spots were used as the training set, while 151509_full_image and its respective spots were used as the test set.
- SpatialLIBD_2 includes a total of 8 whole-slide H&E images, 151669_full_image, 151670_full_image, 151672_full_image, 151673_full_image, 151674_full_image, 151675_full_image, and 151676_full_image, and their respective spots were used as the training set, while 151671_full_image and its respective spots were used as the test set.

G Experimental design for CLIP

In our experiments, we specifically adapted and trained the CLIP architecture for the task of gene expression prediction from histology images. While the original CLIP model was designed for general-purpose image-text contrastive learning, we repurposed its framework to align histology images with gene expression data by treating gene expression profiles as a form of natural language. Specifically, for each spatial spot, we ranked genes by their expression levels in descending order. The top-ranked gene symbols were then concatenated into a string, separated by spaces (e.g., "AAAS AACS AAED1 AAGAB AAK1 AAMDC AAMP AAR2 AARD ...") to serve as the textual input to the CLIP model's language encoder. We preserved the standard CLIP image encoder without modifying the input format but trained the model using paired image–gene expression data under our task setting. The training process follows the original CLIP methodology, with modifications tailored to our application. After training, the model generates embeddings for query image patches and retrieves the top-K most similar reference patches from the training set. The predicted gene expression for a given query patch is obtained by averaging the expression profiles of its top-K nearest neighbors in the reference set.

References

[1] Taku Monjo, Masaru Koido, Satoi Nagasawa, Yutaka Suzuki, and Yoichiro Kamatani. Efficient prediction of a spatial transcriptomics profile better characterizes breast cancer tissue sections without costly experimentation. *Scientific reports*, 12(1):4133, 2022.

[2] Bryan He, Ludvig Bergenstråhle, Linnea Stenbeck, Abubakar Abid, Alma Andersson, Åke Borg, Jonas Maaskola, Joakim Lundeberg, and James Zou. Integrating spatial gene expression and breast tumour morphology via deep learning. *Nature biomedical engineering*, 4(8):827–834, 2020.

[3] Minxing Pang, Kenong Su, and Mingyao Li. Leveraging information in spatial transcriptomics to predict super-resolution gene expression from histology images in tumors. *BioRxiv*, pages 2021–11, 2021.

[4] Danh-Tai Hoang, Gal Dinstag, Leandro C Hermida, Doreen S Ben-Zvi, Efrat Elis, Katherine Caley, Stephen-John Sammut, Sanju Sinha, Neelam Sinha, Christopher H Dampier, et al. Prediction of cancer treatment response from histopathology images through imputed transcriptomics. *Research Square*, pages rs–3, 2023.

[5] Yuansong Zeng, Zhuoyi Wei, Weijiang Yu, Rui Yin, Yuchen Yuan, Bingling Li, Zhonghui Tang, Yutong Lu, and Yuedong Yang. Spatial transcriptomics prediction from histology jointly through transformer and graph neural networks. *Briefings in Bioinformatics*, 23(5):bbac297, 2022.

[6] Yuran Jia, Junliang Liu, Li Chen, Tianyi Zhao, and Yadong Wang. Thitogene: a deep learning method for predicting spatial transcriptomics from histological images. *Briefings in Bioinformatics*, 25(1):bbad464, 2024.

[7] Alec Radford, Jong Wook Kim, Chris Hallacy, Aditya Ramesh, Gabriel Goh, Sandhini Agarwal, Girish Sastry, Amanda Askell, Pamela Mishkin, Jack Clark, et al. Learning transferable visual models from natural language supervision. In *International conference on machine learning*, pages 8748–8763. PMLR, 2021.

[8] Ronald Xie, Kuan Pang, Sai Chung, Catia Perciani, Sonya MacParland, Bo Wang, and Gary Bader. Spatially resolved gene expression prediction from histology images via bi-modal contrastive learning. *Advances in Neural Information Processing Systems*, 36, 2024.

[9] Bo Li, Yong Zhang, Qing Wang, Chengyang Zhang, Mengran Li, Guangyu Wang, and Qianqian Song. Gene expression prediction from histology images via hypergraph neural networks. *Briefings in Bioinformatics*, 25(6):bbae500, 2024.
