# Supplementary figures and images for: HECLIP: histology-enhanced contrastive learning for imputation of transcriptomics profiles
Source: Bioinformatics. 2025 Jun 26;41(7):btaf363. doi: 10.1093/bioinformatics/btaf363 (PMC12362354; doi:10.1093/bioinformatics/btaf363)

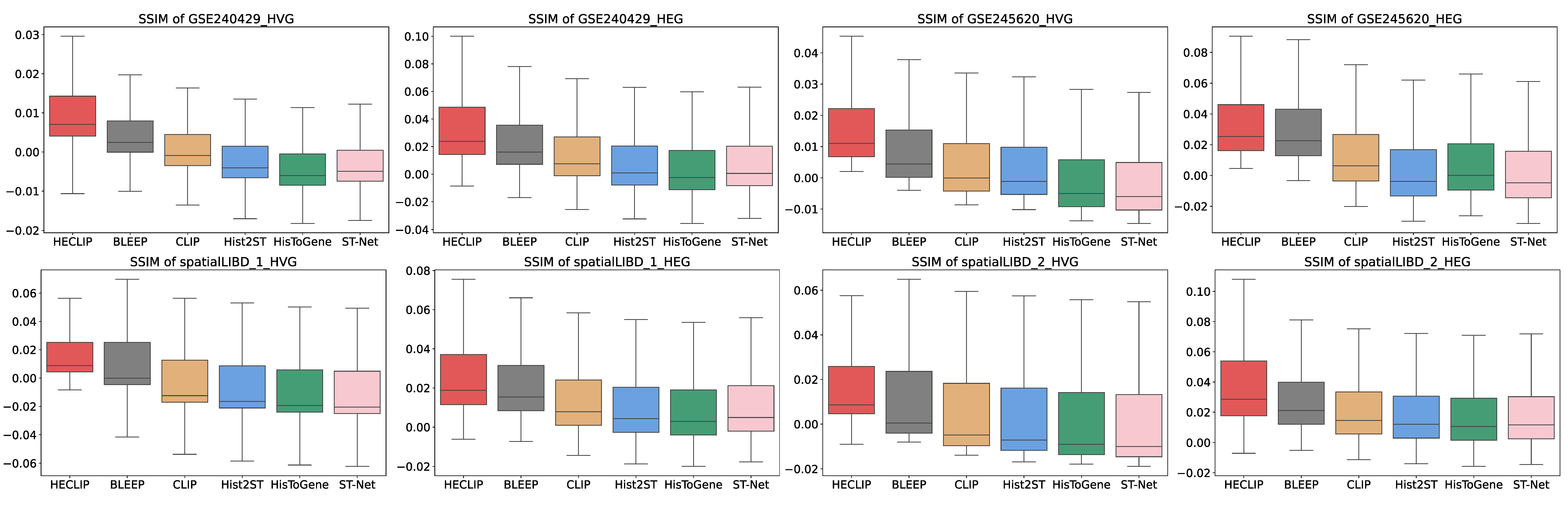

Supplement: btaf363_Supplementary_Data [file btaf363_supplementary_data.zip › Fig S1.png]

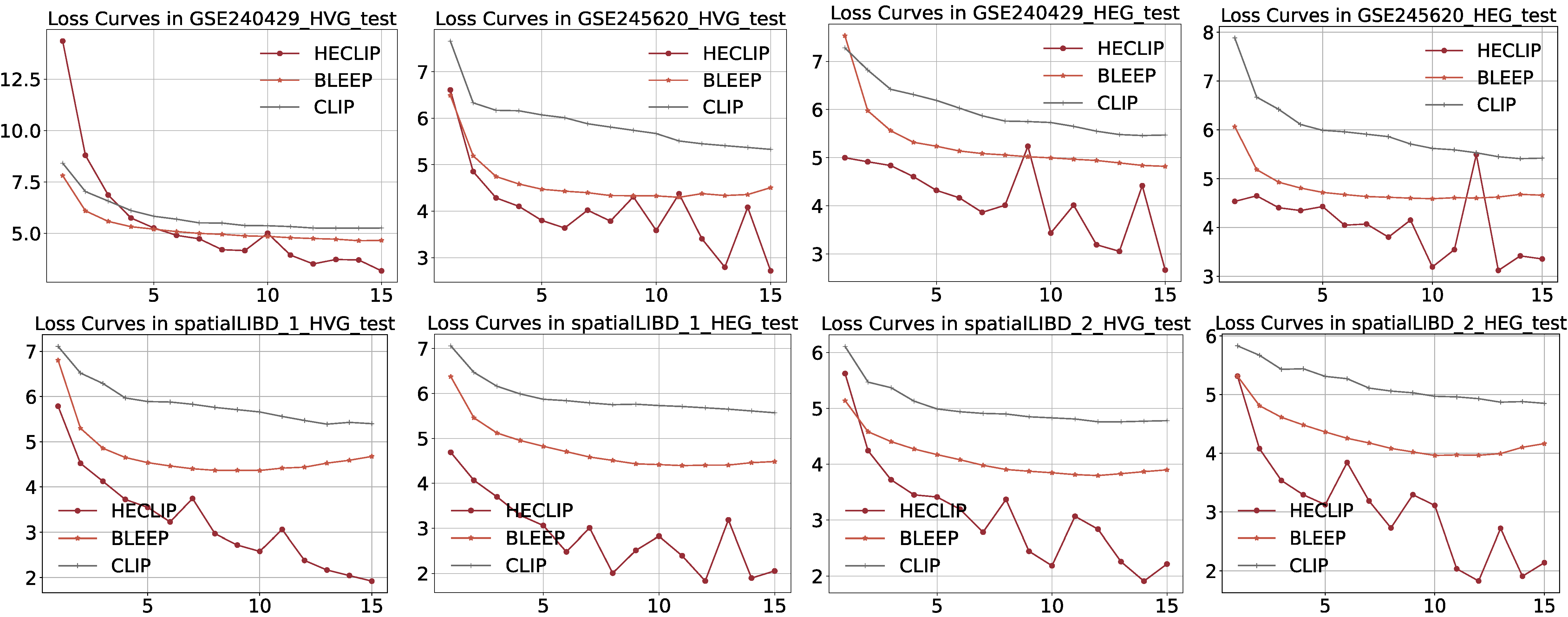

Supplement: btaf363_Supplementary_Data [file btaf363_supplementary_data.zip › Fig S2.png]

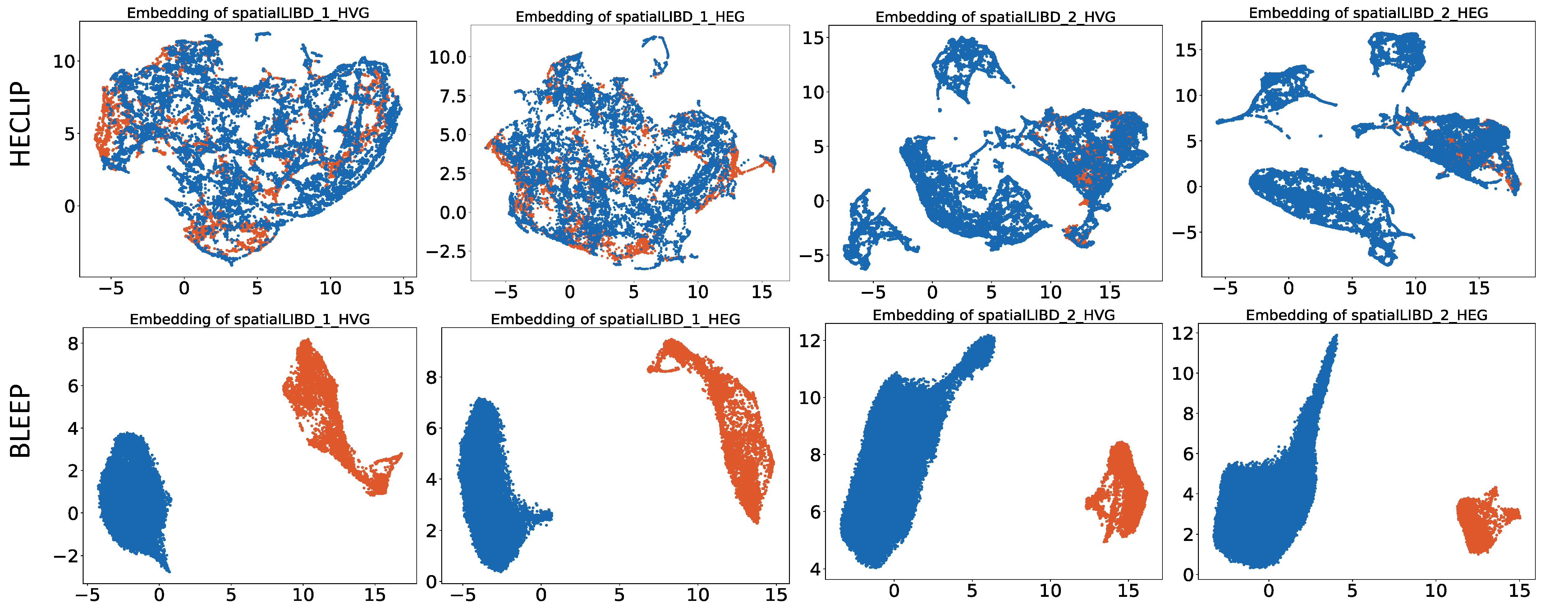

Supplement: btaf363_Supplementary_Data [file btaf363_supplementary_data.zip › Fig S3.png]

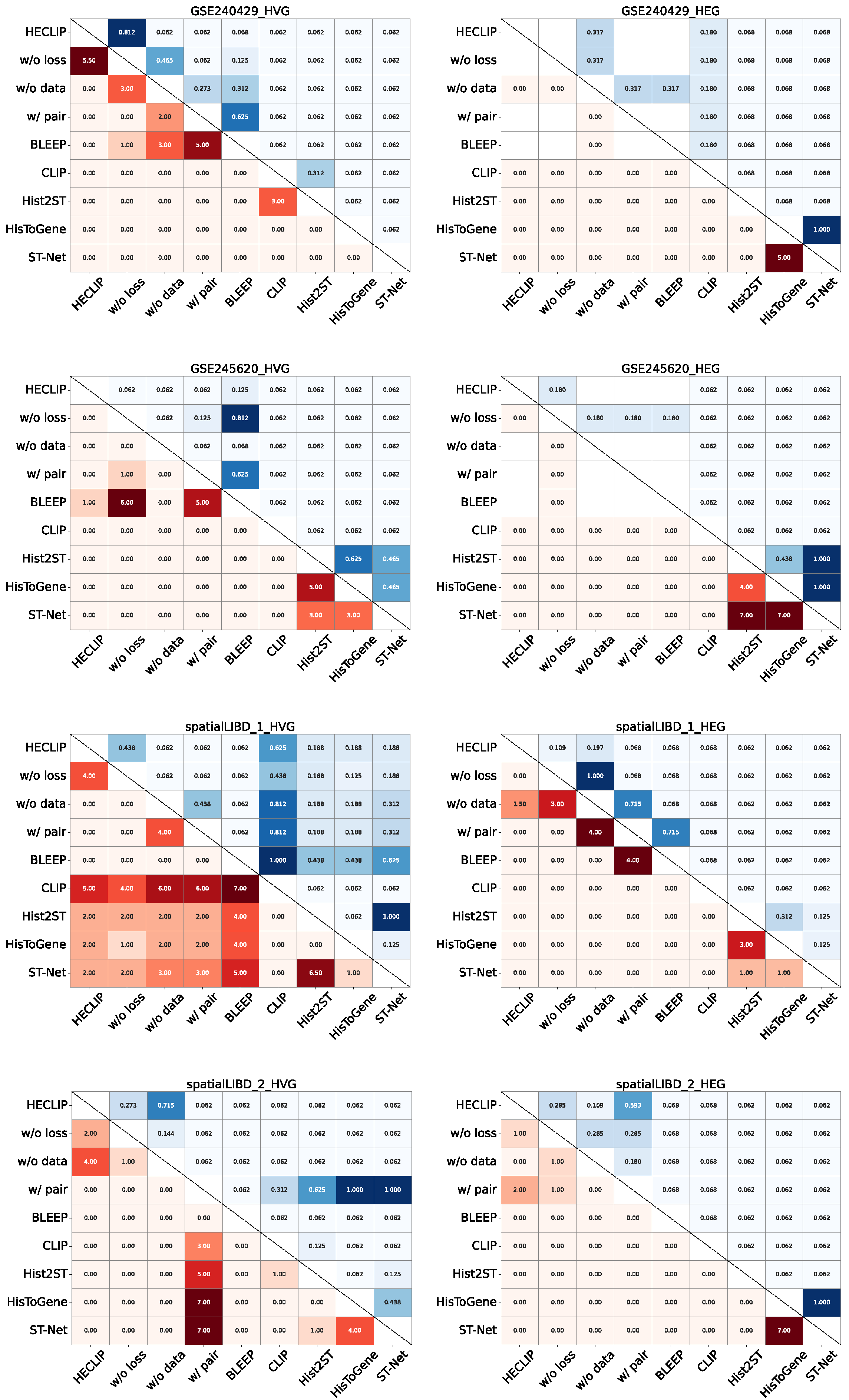

Supplement: btaf363_Supplementary_Data [file btaf363_supplementary_data.zip › Fig S4.png]
